# Supplementary material for: Comparative Efficacy of Intra-Articular Injection, Physical Therapy, and Combined Treatments on Pain, Function, and Sarcopenia Indices in Knee Osteoarthritis: A Network Meta-Analysis of Randomized Controlled Trials
Source: Int J Mol Sci. 2023 Mar 23;24(7):6078. doi: 10.3390/ijms24076078 (PMC10094194; doi:10.3390/ijms24076078)

Supplementary Online Content

Comparative Efficacy of Intra-Articular Injection, Physical Therapy, and Combined Treatments on Pain, Function, and Sarcopenia Indices in Knee Osteoarthritis: A Network Meta-Analysis of Randomized Controlled Trials

Chun-De Liao, Hung-Chou Chen, Mao-Hua Huang, Tsan-Hon Liou, Che-Li Lin, Shih-Wei Huang

Table S1. Database search formulas

Table S2. Summary of included study characteristics

Table S3. Summary of methodological quality based on the PEDro classification scale

Table S4. League Supplementary Table for pairwise and network meta-analysis of mean change in pain score from baseline

Table S5. GRADE certainty ratings for each outcome

Table S6. League Supplementary Table for pairwise and network meta-analysis of mean change in global function from baseline

Table S7. League Supplementary Table for pairwise and network meta-analysis of mean change in walking capability from baseline

Table S8. Associations of moderators with treatment efficiency for all outcome measures

Table S9. Summary of compliance & adverse events

Figure S1. Forest plot of node-splitting results for pain

Figure S2. Forest plot summarizing the effects of treatment regimens on pain reduction for each follow-up time frame

Figure S3. Forest plot of node-splitting results for global function

Figure S4. Forest plot summarizing the effects of treatment regimens on global function recovery for each follow-up time frame

Figure S5. Forest plot of node-splitting results for walking capability

Figure S6. Forest plot summarizing the effects of treatment regimens on walking capability restoration for each follow-up time frame

Figure S7. Funnel plot representing publication bias for pain (A), global function (B), and walking capability (C)

Supplementary Table S1. Database search formulas

Data base	Search terms for query
**Pubmed**	
#1	((knee osteoarthritis) OR gonarthritis) OR knee arthritis
#2	((((((((Injection) OR platelet rich plasma) OR hyaluronic acid) OR corticosteroid) OR autologous conditioned plasma) OR bone marrow aspirate) OR ozone) OR mesenchymal stem cell) OR dextrose prolotherapy) OR botulinum toxin
#3	(((((((physical therapy) OR physiotherapy) OR exercise training) OR physical activity) OR hydrotherapy) OR aquatic therapy) OR neuromuscular training) OR vibration training
#4	(((((physical agent modality) OR electrotherapy) OR shockwave therapy) OR thermal therapy) OR ultrasound) OR neuromuscular electrical stimulation
#5	#3 OR #4
#6	random*:ab,ti OR factorial*:ab,ti OR crossover*:ab,ti OR placebo*:ab,ti OR control*:ab,ti OR trial:ab,ti OR group*:ab,ti OR 'crossover procedure'/exp OR 'single blind procedure'/exp OR 'double blind procedure'/exp OR 'randomised controlled trial'/exp
#7	((((#1) AND 2) AND #5) AND #6
**Physiotherapy Evidence Database (PEDro)**	
	Method: clinical trial
	Abstract & Title:
#1	knee osteoarthritis
#2	Injection OR platelet rich plasma OR hyaluronic acid OR corticosteroid OR autologous conditioned plasma) OR bone marrow aspirate OR ozone OR mesenchymal stem cell OR dextrose prolotherapy OR botulinum toxin
#3	Physical therapy OR physiotherapy OR exercise training OR physical activity OR hydrotherapy OR aquatic therapy OR neuromuscular training OR vibration training
#4	Electrotherapy OR shockwave therapy OR thermal therapy OR ultrasound OR neuromuscular electrical stimulation

(continued)

Supplementary Table S1. (continued)

Data base	Search terms for query
**Excerpta Medica dataBASE (EMBASE)**	
#1	knee osteoarthritis OR gonarthritis OR knee arthritis
#2	Injection OR platelet rich plasma OR hyaluronic acid OR corticosteroid OR autologous conditioned plasma) OR bone marrow aspirate OR ozone OR mesenchymal stem cell OR dextrose prolotherapy OR botulinum toxin
#3	Physical therapy OR physiotherapy OR exercise training OR physical activity OR hydrotherapy OR aquatic therapy OR neuromuscular training OR vibration training
#4	Electrotherapy OR shockwave therapy OR thermal therapy OR ultrasound OR neuromuscular electrical stimulation
#5	#3 OR #4
#6	#1 AND #2 AND #5 AND ([systematic review]/lim OR [meta analysis]/lim OR [randomized controlled trial]/lim) AND [humans]/lim
**Cochrane Library Database**	
#1	knee osteoarthritis OR gonarthritis OR knee arthritis
#2	Injection OR platelet rich plasma OR hyaluronic acid OR corticosteroid OR autologous conditioned plasma OR bone marrow aspirate OR ozone OR mesenchymal stem cell OR dextrose prolotherapy OR botulinum toxin
#3	Physical therapy OR physiotherapy OR exercise training OR physical activity OR hydrotherapy OR aquatic therapy OR neuromuscular training OR vibration training
#4	Electrotherapy OR shockwave therapy OR thermal therapy OR ultrasound OR neuromuscular electrical stimulation
#5	randomized controlled trial OR randomised controlled trial
#6	#1 AND #2 AND (#3 OR #4) AND #5

(continued)

Supplementary Table S1. (continued)

Data base	Search terms for query
**China knowledge resource integrated database**	
#1	knee osteoarthritis OR gonarthritis OR knee arthritis
#2	Injection OR platelet rich plasma OR hyaluronic acid OR corticosteroid OR autologous conditioned plasma OR bone marrow aspirate OR ozone OR mesenchymal stem cell OR dextrose prolotherapy OR botulinum toxin
#3	Physical therapy OR physiotherapy OR exercise training OR physical activity OR hydrotherapy OR aquatic therapy OR neuromuscular training OR vibration training
#4	Electrotherapy OR shockwave therapy OR thermal therapy OR ultrasound OR neuromuscular electrical stimulation
#5	randomized controlled trial OR randomised controlled trial
#6	#1 AND #2 AND (#3 OR #4) AND #5
**Google Scholar**	
#1	allintitle: knee osteoarthritis
#2	allintitle: injection OR platelet rich plasma OR hyaluronic acid OR corticosteroid OR autologous conditioned plasma OR bone marrow aspirate OR ozone OR mesenchymal stem cell OR dextrose prolotherapy OR botulinum toxin
#3	allintitle: “physical therapy” OR physiotherapy OR exercise training OR physical activity OR hydrotherapy OR aquatic therapy OR neuromuscular training OR vibration training OR electrotherapy OR shockwave therapy OR thermal therapy OR ultrasound OR “neuromuscular electrical stimulation”
#4	allintitle: randomized controlled trial OR randomised controlled trial

Supplementary Table S2. Summary of included study characteristics

Study, (year)[Referenae]	Country (area)	Study arm	Age (years)	BMI (kg/m²)	Female	*N*	OA dignosis	Involved side	K-L grade	Disease duration (months)	Co-intervention	Measured time point (week)	Main outcome measures	Injection protocol		
			Mean (SD or range)	Mean (SD or range)	n (%)			Unilateral /Bilateral		Mean (SD, range)				Injection time (protocol)	Time interval per injection (week)	No. of injections
Acosta-Olivo 2014	Mexico	Gr 1: PRP + EX	>40[a]	NR	NR	21	Radiographic	NR	I, II	≥3	None	0, 4, 16, 24	KOOS	0, 2	2	2
	(America)	Gr 2: EX				21										
Akan 2018	Turkey	Gr 1: PRP + EX	65.0 (7.8)	33.6 (5.2)	24 (80)	30	ACR	NR	IV	NR	None	0, 12, 24	VAS; WOMAC	0, 3, 6	3	3
	(Asia)	Gr 2: EX	56.3 (10.3)	32.7 (2.1)	29 (96.7)	30										
Altman 2009	USA	Gr 1: HA + PT	62.5 (11.0)	32.4 (7.0)	184 (63.2)	291	ACR	291/0	II, III	NR	Analgesics;	0, 1, 2, 3, 6,	VAS; WOMAC	0, 1, 2	1	3
	(America)	Gr 2: PLA + PT	60.8 (10.0)	33.0 (7.0)	186 (63.1)	295		295/0			corticosteroids	12, 18, 26	50-foot walk			
Angoorani 2015	Iran	Gr 1: PRP	62.2 (12.1)	28.5 (3.8)	22 (81.5)	27	ACR	NR	I-III	NR	paracetamol	0, 4, 8	VAS	0, 4	4	2
	(Asia)	Gr 2: PT	61.6 (8.1)	29.2 (3.2)	25 (92.6)	27							KOOS			
Anz 2020	USA	Gr 1: MSC+PT	55.8 (11.3)	27.7 (5.0)	18 (40)	45	Radiographic	NR	I-III	≥4.0	None	0, 4, 12, 24,	WOMAC	0	0	1
	(America)	Gr 2: PRP+PT	52.2 (12.4)	27.9 (5.8)	17 (43.6)	39						36, 52		0	0	1
Atamaz 2006	Turkey	Gr 1: HA	62.4 (9.0)	30.1 (5.2)	18 (90)	20	ACR	20/0	II, III	≥6	Paracetamol	0, 4, 12, 24,	VAS; WOMAC	0, 1, 2, 24		4
	(Asia)	Gr 2: HA	60.4 (9.3)	29.9 (2.7)	15 (75)	20		20/0				36, 52	15-m walk			
		Gr 3: PTA	58.7 (8.3)	30.9 (2.3)	34 (81)	42		40/0								
Auerbach 2002	Germeny	Gr 1: HA+EX	48 (17-78)	NR	29 (51.8)	56	Radiographic	56/0	I-IV	NR	None	0, 52	VAS; WOMAC	0, 1, 2	1	3
	(Europe)	Gr 2: OZ+EX	46.5 (18-80)		27 (50.9)	53		53/0							3	5
Babaei-Ghazani 2018	Iran	Gr 1: CS+EX	56.3 (7.9)	29.2 (4.5)	28 (90.3)	31	ACR	31/0	I-III	67.0 (18.5)	None	0, 1, 4, 12	VAS; WOMAC	0	0	1
	(Asia)	Gr 2: OZ + EX	59.7 (10.2)	28.8 (2.5)	24 (77.4)	31		31/0		67.3 (15.7)						
Babaei-Ghazani 2019	Iran	Gr 1: CS + EX	58.5 (9.2)	29.2 (4.4)	14 (87.5)	16	ACR	16/0	III	67.0 (18.5)	None	0, 1, 4, 12	VAS; WOMAC	0	0	1
	(Asia)	Gr 2: OZ + EX	60.6 (9.6)	28.6 (5.4)	11 (68.8)	16		16/0		67.3 (15.7)						
Bao 2018	China	Gr 1: HA + EX	66.0 (2.1)	NR	7 (35)	20	Radiographic	20/0	II-IV	31.8 (6.11)	None	0, 4, 8	VAS; WOMAC	0, 1, 2, 3, 4	1	5
	(Asia)	Gr 2: BoNTA + EX	66.4 (3.5)		10 (50)	20		20/0		30.2 (7.93)				0	0	1
		Gr 3: PLA + EX	65.3 (3.5)		11 (55)	20		20/0		33.6 (9.39)						
Baranova 2018	Ukraine	Gr 1: OZ + PTA	42.0 (5.0)[a]	NR	29 (48.3)	45	Radiographic	NR	I, II	48.0 (14.4)[a]	Drug medicine	0, 4, 24	VAS; WOMAC	1 injection/day	1 (day)	10
	(Europe)	Gr 2: PTA				44										
Basar 2021	Turkey	Gr 1: HA + PT	49.9 (5.0)	28.3 (2.1)	20 (64.5)	31	Radiographic	31/0	I-III	40.72 (15.14)	TENS	0, 4, 24	VAS; WOMAC	0	0	1
	(Asia)	Gr 2: PT	50.9 (4.5)	28.7 (2.2)	29 (64.4)	45		45/0		39.43 (12.34)						
Baygutalp 2021	Turkey	Gr 1: DxTP + EX	56.6 (7.1)	34.3 (4.6)	21 (84)	25	ACR	25/0	II, III	35.1 (29.6)	None	0, 6, 12	VAS	0, 3, 6	3	3
	(Asia)	Gr 2: OZ + EX	57.0 (7.6)	33.2 (4.4)	22 (88)	25		25/0		34.3 (27.6)			WOMAC	0, 1, 2	1	3
		Gr 3: EX	56.5 (7.4)	32.2 (5.4)	21 (84)	25		25/0		30.8 (31.9)			TUG;			

To be continued.

Supplementary Table S2. Continued

Study	Country	Study arm	Age (years)	BMI (kg/m^2)	Female	*N*	OA dignosis	Involved side	K-L grade	Disease duration (months)	Co-intervention	Measured time point (week)	Main outcome measures	Injection protocol		
(year)[Referenae]	(area)		Mean (SD or range)	Mean (SD or range)	n (%)			Unilateral /Bilateral		Mean (SD, range)				Injection time (protocol)	Time interval per injection (week)	No. of injections
Bayramoglu 2003	Brazil	Gr 1: HA	62.6 (10.2)	NR	35 (94.6)	16	Symptomatic	4/12	NR	79.2 (62.4)	None	0, 3, 12	ISK	0, 1, 2	1	3
	(Europe)	Gr 2: HA	61.5 (10.9)			12		4/8		63.6 (56.4)				0, 1, 2	1	3
		Gr 3: PT	60.7 (8.5)			9		0/9		64.8 (74.4)						
Centeno 2018	USA	Gr 1: MSC + EX	57.0 (8.5)	26.0 (2.9)		22	Symptomatic;	NR	II, III	NR	None	0, 6, 12,	VAS;	0	0	1
	(America)	Gr 2: MSC	54.0 (8.9)	26.0 (2.9)		26	radiographic					24, 52, 144	KSS			
		Gr 3: EX	57.0 (8.5)	26.0 (2.9)		22										
Chen 2013	Taiwan	Gr 1: HA	67.9 (9.9)	27.1 (5.3)[b]	23 (85.2)	27	ACR	27/0	II-IV	84.5 (68.4)	None	0, 6, 12, 16	VAS; 30-m walk	0, 1, 2, 3, 4	1	5
	(Asia)	Gr 2: PTA	66.5 (7.2)	24.4 (4.1)[b]	20 (87)	23		23/0		16.9 (27.9)			Lequesne index			
Cole 2017	USA	Gr 1: HA + EX	56.8 (10.5)	29.0 (6.4)	30 (60)	50	Radiographic	50/0	I-III	NR	None	0, 2, 3, 6, 12,	VAS	0, 1, 2	1	3
	(America)	Gr 2: PRP + EX	55.9 (10.4)	27.4 (3.9)	21 (42.9)	49		49/0				24, 52	IKDC	0, 1, 2	1	3
de Sire 2020	Italy	Gr 1: HA + EX	70.7 (5.4)	26.8 (1.7)	13 (65)	20	Radiographic	NR	II, III	NR		0, 1, 2, 3,	VAS;	0, 1, 2, 3	1	4
	(Europe)	Gr 2: OZ + EX	70.3 (6.5)	27.1 (1.9)	16 (72.7)	22						7, 24	OKS	0, 1, 2, 3	1	4
DeCaria 2012	Canada	Gr 1: HA + EX	71.9 (6.8)	30.5 (6.2)	7 (46.7)	15	ACR	5/10	II, III	NR	Acetaminophen	0, 4, 12,	WOMAC	0, 1, 2	1	3
	(America)	Gr 2: PLA + EX	72.9 (5.5)	29.4 (4.1)	7 (46.7)	15		6/9				24	20-m walk			
Delgado-Enciso 2018	Mexico	Gr 1: CS + PT	68.5 (8.9)	28.0 (3.3)	4 (50)	8	ACR	8/0	IV	≥6.0	paracetamol/NSAID	0, 12, 24,	WOMAC	0, 12, 24	12	3
	(America)	Gr 2: PT	68.0 (7.1)	29.8 (2.1)	3 (37.5)	8		8/0				52				
Delgado-Enciso 2019	Mexico	Gr 1: CS + PT	60.7 6.7)	32.7 (3.3)	62 (57.9)	107	ACR	NR	IV	≥6.0	paracetamol/NSAID	0, 24, 52	VAS	0, 4, 8	4	3
	(America)	Gr 2: PT	61.5 (8.2)	31.9 (4.0)	63 (60)	105										
Deyle 2020	USA	Gr 1: CS	56.0 (8.2)	31.6 (6.1)	38 (48.7)	78	ACR	29/49	I-IV	85.0 (89.2)		0, 4, 8, 24,	WOMAC	0, 16, 36	16	1-3
	(America)	Gr 2: PT	56.3 (9.2)	31.4 (5.1)	37 (47.4)	78		29/49		100 (122.7)		52	TUG			
Di Sante 2012	Italy	Gr 1: CS	70.5 (7.6)	NR		20	ACR	NR	II, III	NR	None	0, 1, 4	VAS	0	0	1
	(Europe)	Gr 2: PTA	70.8 (7.4)			20							WOMAC			
		Gr 3: CS + PTA	70.5 (8.3)			20										
Dumais 2012	Canada	Gr 1: DxTP + EX	57.3 (12.6)	32.2 (7.2)	7 (38.9)	18	Radiographic	NR	I-IV	≥6.0	None	0, 4, 8, 12,	WOMAC	0, 4, 8, 12	4	4
	(America)	Gr 2: EX	56.2 (10.9)	34.3 (5.7)	10 (55.6)	18						16, 20	TUG			
Elerian 2016	Egypt	Gr 1: CS	51.0 (3.5)[a]	NR	50 (83.3)	20	ACR	0/20	II, III	NR	None	0, 4, 8, 24	VAS	0, 4	4	2
	(Africa)	Gr 2: PTA (ESWT)				20		0/20					WOMAC			
		Gr 3: UC (shame ESWT)				20		0/20								
Elgendy 2020	Egypt	Gr 1: PRP + PT	49.2 (9.2)	31.5 (1.9)		15	ACR	15/0	II, III	NR	None	0, 4	VAS; WOMAC	0	0	1
	(Africa)	Gr 2: PT	51.9 (8.2)	31.1 (2.3)		30		30/0								
Elik 2020	Turkey	Gr 1: PRP + EX	61.3 (7.9)	30.4 (4.5)	29 (96.7)	30	ACR	30/0	I-III	≥12	Paracetamol;	0, 4, 24	VAS; WOMAC	0, 1, 2	1	3
	(Asia)	Gr 2: PLA + EX	60.2 (6.8)	30.7 (4.0)	24 (88.9)	27		27/0			NSAIDs					

To be continued.

Supplementary Table S2. Continued

Study	Country	Study arm	Age (years)	BMI (kg/m²)	Female	*N*	OA dignosis	Involved side	K-L grade	Disease duration (months)	Co-intervention	Measured time point (week)	Main outcome measures	Injection protocol		
(year)[Referenae]	(area)		Mean (SD or range)	Mean (SD or range)	n (%)			Unilateral /Bilateral		Mean (SD, range)				Injection time (protocol)	Time interval per injection (week)	No. of injections
Filardo 2015	Italy	Gr 1: HA + PA	57.6 (11.8)	26.9 (4.4)	37 (41.6)	89	Radiographic	89/0	I-III	68.4 (54-300)	None	0, 10, 26,	VAS;	0, 1, 2	1	3
	(Europe)	Gr 2: PRP + PA	53.3 (13.2)	26.6 (4.0)	34 (36.2)	94		94/0		65.5 (54-360)		52	KOOS	0, 1, 2	1	3
Forogh 2016	Iran	Gr 1: CS + EX	61.1 (6.7)	29.2 (3.4)	15 (62.5)	24	ACR	24/0	II, III	≥3	None	0, 8, 24	VAS; KOOS	0	0	1
	(Asia)	Gr 2: PRP + EX	59.1 (7.0)	28.9 (2.8)	17 (70.8)	24		24/0					20-m walk	0	0	1
Freitag 2019	Australia	Gr 1: 1-injection MSC + EX	54.6 (6.3)	31.6 (5.9)	3 (30)	10	ACR	10/0	II, III	NR	None	0, 4, 12,	VAS; WOMAC	0	0	1
	(Oceania)	Gr 2: 2-injection MSC + EX	54.7 (10.2)	30.4 (5.6)	6 (60)	10		10/0				24, 52	KOOS	0, 24	24	2
		Gr 3: EX	51.5 (6.1)	25.2 (3.4)	5 (50)	10		10/0								
Gaballa 2019	Egypt	Gr 1: PRP	53.6 (4.6)	NR	15 (75)	20	ACR	20/0	I-III	64.8 (38.4)	None	0, 4, 12	VAS	0, 2	2	2
	(Africa)	Gr 2: OZ	56.3 (4.4)		16 (80)	20		20/0		70.8 (42.0)			WOMAC	0, 1, 2, 3	1	4
		Gr 3: EX + PTA	55.3 (4.2)		15 (75)	20		20/0		76.8 (39.6)			6MWD			
García-Triana 2021	Mexico	Gr 1: DxTP + PT	55.5 (14.8)	NR	18 (72)	25	Radiographic	1/24	I-III	NR	None	0, 24	VAS; WOMAC	0, 1, 2, 3, 4	1	5
	(America)	Gr 2: PT	53.5 (14.4)		20 (80)	25		0/25								
Garza 2020	USA	Gr1: High-dose SVF + PA	59.5 (11.7)	28.8 (4.3)	6 (46.2)	13	Radiographic	13/0	II, III	≥3	None	0, 6, 12, 24, 52	WOMAC	0	0	1
	(America)	Gr2: Low-dose SVF + PA	60.5 (7.9)	27.6 (4.1)	9 (69.2)	13		13/0						0	0	1
		Gr3: PLA + PA	57.1 (9.1)	27.1 (2.7)	7 (53.8)	13		13/0								
Ghai 2019	India	Gr 1: PRP + EX	49.8 (9.4)[a]	NR	15 (37.5)	20	Radiographic	20/0[a]	I, II	≥4.0	Acetaminophen;	0, 2, 6,	VAS	0	0	1
	(Asia)	Gr 2: PLA + EX				20					oral tramadol	12, 24	WOMAC			
Hawkins 2012	UK	Gr 1: CS	58.0 (11.1)	NR	9 (60)	15	Radiographic	15/0	II, III	NR	None	0, 2, 6, 12	WOMAC	0	0	1
	(Europe)	Gr 2: CS + EX	63.0 (7.1)		9 (52.9)	17		17/0								
Henriksen 2015	Denmark	Gr 1: CS + EX	61.3 (9.9)	29.0 (3.9)	28 (56)	50	Radiographic	NR	I-IV	88.8 (9.6-228)	None	0, 2, 14, 26	KOOS; 6MWD	0	0	1
	(Europe)	Gr 2: PLA + EX	65.5 (8.3)	28.9 (3.3)	33 (66)	50				91.2 (6.0-240)						
Hermans 2019	Hermans	Gr 1: HA + PT	53.6 (8.6)	28.9 (5.2)	37 (48.1)	77	Radiographic	NR	I-III	>3	acetaminophen or	0, 52	VAS	0, 1, 2	1	3
	(Europe)	Gr 2: PT	54.8 (6.4)	29.2 (5.4)	40 (50.6)	79					NSAIDs		KOOS			
Huang 2005	Taiwan	Gr 1: EX	65.0 (6.4)[a]	NR	113 (27.1)	35	NR	0/35	III	5.0-144.0	None	0, 8, 52	VAS;	0, 1, 2, 3, 4	1	5
	(Asia)	Gr 2: EX + PTA				35		0/35					GS			
		Gr 3: HA + EX+PTA				35		0/35					Lequesne's index			
		Gr 4: UC				35		0/35								
Ip 2015	China	Gr 1: HA + PT	75 (70–80)[a]	NR	50 (35.7)	70	Radiographic	0/70[a]	III	NR	None	0, 84	WOMAC	0, 1, 2, 3, 4	1	5
	(Asia)	Gr 2: PLA + PT				70										

To be continued.

Supplementary Table S2. Continued

Study	Country	Study arm	Age (years)	BMI (kg/m^2)	Female	*N*	OA dignosis	Involved side	K-L grade	Disease duration (months)	Co-intervention	Measured time point (week)	Main outcome measures	Injection protocol		
(year)[Referenae]	(area)		Mean (SD or range)	Mean (SD or range)	n (%)			Unilateral /Bilateral		Mean (SD, range)				Injection time (protocol)	Time interval per injection (week)	No. of injections
Isik 2020	Turkey	Gr 1: DxTP + PTA	62.8 (7.5)	23.7 (2.8)	17 (54.8)	31	Radiographic	14/17	II, III	44 (6–180)	None	0, 6, 12	VAS; WOMAC	0, 3, 6	3	3
	(Asia)	Gr 2: DxTP	58.7 (8.2)	24.5 (3.2)	19 (59.4)	32		16/16		52 (10–144)						
Jhan 2022	Taiwan	Gr 1: HA + PT	52 (33-64)	NR	13 (86.7)	15	Radiographic	13/2	I, II	NR	None	0, 12, 24,	VAS; WOMAC	0, 1, 2	1	3
	(Asia)	Gr 2: PT	54 (40-60)		14 (93.3)	15		14/1				48	KOOS			
		Gr 3: UC	49 (30-63)		14 (93.3)	15		13/2								
Karatosun 2006	Turkey	Gr 1: HA	57.8 (12.1)	27.9 (5.6)	44 (84.6)	52	ACR	10/95[a]	III	NR	None	0, 1, 2, 3, 6,	VAS	0, 1, 2	1	3
	(Asia)	Gr 2: EX	55.3 (13.6)	28.3 (4.9)	46 (86.8)	53						12, 24, 52, 72	HSS			
Kaszyński 2022	Poland	Gr 1: MSC + EX	55.0 (8.0)	27.0 (3.0)		20	Symptomatic	20/0	I-III	NR	None	0, 4, 12, 24,	VAS; WOMAC	0, 1, 2	1	3
	(Europe)	Gr 2: PRP + EX	57.0 (8.0)	26.0 (3.0)		20		20/0				52	TUG; 10-m walk	0	0	1
Kawasaki 2009	Japan	Gr 1: HA	69.5 (8.4)	25.7 (4.1)	42 (100)	42	ACR	NR	II, III	2.0-24.0[a]	Sodium loxoprofen	0, 24	VAS;	0, 1, 2, 3, 4, 8,	1–4	11
	(Asia)	Gr 2: EX	71.2 (7.1)	24.6 (3.0)	45 (100)	45					tablets		JKOM	12, 16,20, 24		
Khalifeh Soltani 2019	Iran	Gr 1: MSC + PA	57.5	29.6	9 (90)	10	Radiographic	NR	II-IV	NR	acetaminophen	0, 2, 8, 24	VAS;	0	0	1
	(Asia)	Gr 2: PLA + PA	55.8	28.9	9 (90)	10							KOOS			
Kon 2018	Italy	Gr 1: ACS + PA	57 (41-68)	NR	13 (41.9)	31	Radiographic	NR	II, III	≥6.0	acetaminophen/	0, 2, 4, 12,	VAS	0	0	1
	(Europe)	Gr 2: UC (PLA)	54 (44-67)		6 (40)	15					paracetamol	24, 52, 144	WOMAC			
Kon 2020	Italy	Gr 1: ACS + PA	54 (44-67)	NR	6 (42.9)	14	Radiographic	NR	II, III	≥6.0	acetaminophen/	0, 52, 104	VAS; WOMAC	0	0	1
	(Europe)	Gr 2: UC	57 (41-68)		13 (44.8)	29					paracetamol		KOOS			
Lee 2017	Korea	Gr 1: HA + PA	69.1 (6.2)	24.2 (3.1)	4 (13.3)	30	ACR	NR	II, III	19.1 (12.8)	None	0, 4, 12	VAS; WOMAC	0, 1, 2	1	3
	(Asia)	Gr 2: ESWT + PA	67.7 (5.5)	24.9 (3.9)	6 (19.4)	31				17.0 (11.3)			10-m walk			
Liu 2019	China	Gr 1: HA	60.2 (4.2)	24.8 (2.5)	30 (76.9)	39	ACR	NR	I, II	NR	None	0, 5	VAS	0, 1, 2, 3, 4	1	5
	(Asia)	Gr 2: PTA	59.8 (4.4)	25.4 (2.9)	26 (68.4)	38							WOMAC			
Lucangeli 2001	Italy	Gr 1: HA + PT	65.2 (7.4)	NR		10	Radiographic	NR	II, III	NR	None	0, 5, 17, 29	VAS	0, 1, 2, 3, 4	1	5
	(Europe)	Gr 2: PT	69.6 (7.5)			10							Lequesne Index			
McAlindon 2018	USA	Gr 1: BoNTA (high dose) + PT	60.7 (8.3)	31.6 (6.5)	30 (68.2)	44	ACR	29/15	II, III	110.4 (88.8)	acetaminophen	0, 1, 4, 8, 12,	VAS	0	0	1
	(America)	Gr 2: BoNTA (low dose) + PT	60.2 (8.4)	32.3 (7.7)	26 (60.5)	43		31/12		93.6 (92.4)		16, 24, 24	WOMAC	0	0	1
		Gr 3: PLA + PT	61.1 (7.8)	30.4 (5.1)	51 (57.3)	89		57/32		91.2 (68.4)						
Nishida 2021	Japan	Gr 1: HA + PT	63.2 (8.6)	25.6 (3.35)	61 (70.1)	87	ACR	NR	II, III	41.0 (53.1)	acetaminophen	0, 1, 2, 4, 6,	VAS;	0, 4, 8	4	3
	(Asia)	Gr 2: PLA + PT	65.3 (8.1)	24.8 (3.7)	68 (76.4)	89				46.5 (56.1)		8, 10, 12, 16,	WOMAC			
												20, 24				
Paker 2006	Turkey	Gr 1: HA	59.1 (5.3)	28.3 (3.6)[b]		25	Radiographic	NR	II, III	56.5 (70.3)	None	0, 4, 8, 24	WOMAC	0, 1, 2	1	3
	(Asia)	Gr 2: PTA	58.8 (5.1)	30.3 (4.6)[b]		27				42.5 (50.6)			Lequesne Index			

To be continued.

Supplementary Table S2. Continued

Study	Country	Study arm	Age (years)	BMI (kg/m²)	Female	*N*	OA dignosis	Involved side	K-L grade	Disease duration (months)	Co-intervention	Measured time point (week)	Main outcome measures	Injection protocol		
(year)[Referenae]	(area)		Mean (SD or range)	Mean (SD or range)	n (%)			Unilateral /Bilateral		Mean (SD, range)				Injection time (protocol)	Time interval per injection (week)	No. of injections
Paolucci 2021	Italy	Gr 1: OZ + PTA+EX	64.5 (5.5)	25.5 (3.3)	19 (76)	25	ACR	25/0	II, III	>1.5	None	0, 3, 7	VAS	0, 1, 2	1	3
	(Europe)	Gr 2: OZ	59.3 (11.6)	28.8 (2.4)	19 (79.2)	24		24/0					KOOS			
Parfitt 2006	UK	Gr 1: CS + EX	76.1 (7.7)	NR	7 (87.5)	8	Radiographic	NR	NR	10.3 (7.0)	None	0, 8	VAS; WOMAC	0	0	1
	(Europe)	Gr 2: CS	67.2 (6.0)		3 (60)	5				11.6 (10.2)			15-m walk			
Paterson 2016	Australia	Gr 1: HA + PA	52.7 (10.3)	30.9 (5.6)	3 (30)	10	ACR	NR	II, III	NR	paracetamol	0, 4, 12	VAS	0, 1, 2	1	3
	(Oceania)	Gr 2: PRP + PA	49.9 (13.7)	27.9 (11.9)	3 (27.3)	11							KOOS	0, 1, 2	1	3
Petrella 2002	Canada	Gr 1: HA + EX	66.2 (9.2)	30.6 (5.4)	22 (40.7)	54	Radiographic	54/0	I-III	NR	NSAID	0, 4, 12	WOMAC	0, 1, 2	1	3
	(America)	Gr 2: EX	66.3 (8.8)	29.4 (6.3)	11 (42.3)	26		26/0					40-m walk			
		Gr 3: UC (PLA)	62.6 (9.5)	32.7 (4.8)	12 (42.9)	28		28/0								
Qamar 2021	Pakistan	Gr 1: PRP + PT	60.0 (4.7)	29.7 (4.7)	33 (66)	50	Radiographic	36/14	I-III	111.6 (43.2)	paracetamol	0, 4, 12, 24	VAS	0, 1, 2	1	3
	(Asia)	Gr 2: PLA + PT	58.7 (3.9)	31.2 (6.7)	30 (60)	50		36/14		102 (37.2)						
Rabago 2013	USA	Gr 1: DxTP	56.8 (7.9)	NR	19 (63.3)	30	ACR	NR	I-IV	79.8 (62.9)	acetaminophen;	0, 5, 9, 12,	VAS	0, 1, 5, 9, 11, 13	2–4	3–5
	(America)	Gr 2: EX	56.4 (7.0)		21 (67.7)	31				60.4 (71.6)	oxycodone tablets	24, 52	WOMAC			
		Gr 3: UC	56.8 (6.7)		20 (69.0)	29				108 (99.5)						
Raeissadat 2015	Iran	Gr 1: HA + EX	61.1 (7.5)	28.6 (1.7)	47 (75.8)	62	ACR	NR	II, III	≥3	paracetamol	0, 52	WOMAC	0, 1, 2	1	3
	(Asia)	Gr 2: PRP + EX	56.9 (9.1)	26.8 (2.0)	69 (89.6)	77								0, 4	4	2
Raeissadat 2018	Iran	Gr 1: HA + EX	61.1 (6.4)	28.6 (1.7)	56 (75.7)	74	ACR	NR	II, III	≥6.0	paracetamol	0, 24	VAS; WOMAC	0, 1, 2	1	3
	(Asia)	Gr 2: OZ + EX	58.1 (6.4)	26.8 (2.0)	50 (74.6)	67								0, 1, 2	1	3
Raeissadat 2020a	Iran	Gr 1: PRP + EX	57.6 (5.9)[a]	28.5 (3.2)[a]	21 (100)	21	ACR	0/21	I-III	67.7 (39.6)	paracetamol	0, 32	VAS; WOMAC	0, 4	4	2
	(Asia)	Gr 2: EX			21 (100)	21		0/21		79.3 (56.4)						
Raeissadat 2020b	Iran	Gr 1: HA + EX	58.6 (7.1)	28.7 (3.0)	37 (71.2)	52	ACR	52/0	II, III	67.7 (39.6)	acetaminophen	0, 8, 24, 52	VAS; WOMAC	0, 1, 2	1	3
	(Asia)	Gr 2: PRGF + EX	57.1 (7.3)	27.9 (2.7)	36 (72)	50		50/0		79.3 (56.4)			Lequesne Index	0, 3	3	2
Raeissadat 2021	Iran	Gr 1: HA + EX	57.9 (6.7)	27.5 (2.2)	37 (75.5)	49	ACR	49/0	II, III	46.3 (19.2)	paracetamol	0, 8, 24, 52	VAS; WOMAC	0, 1, 2	1	3
	(Asia)	Gr 2: PRP + EX	56.1 (6.0)	27.4 (2.6)	39 (75)	52		52/0		53.3 (27.6)			Lequesne Index	0, 3	3	2
		Gr 3: PRGF + EX	56.1 (6.3)	27.5 (2.1)	37 (72.5)	51		51/0		58.8 (32.4)				0, 3	3	2
		Gr 4: OZ + EX	57.6 (6.1)	27.0 (1.9)	36 (75)	48		48/0		53.0 (25.2)				0, 1, 2	1	3
Rayegani 2014	Iran	Gr 1: PRP + EX	58.1 (8.9)	28.2 (4.1)	29 (93.5)	31	ACR	31/0	I-IV	≥3	acetaminophen	0, 8, 12, 28	WOMAC	0, 4	4	2
	(Asia)	Gr 2: EX	54.7 (10.8)	27.3 (3.3)	29 (93.5)	31		31/0								
Raynauld 2002	Canada	Gr 1: HA + PT	62.6 (9.4)	32.1 (8.0)	86 (67.7)	127	Radiographic	18/109	I-IV	108.0 (114.0)	Pain medications	0, 4, 8, 16,	WOMAC	0, 1, 2	1	3
	(America)	Gr 2: PT	63.5 (10.5)	32.9 (7.2)	93 (72.7)	128		20/108		118.8 (116.4)		24, 32, 40, 52				
Rezasoltani 2020	Iran	Gr 1: BoNTA + EX	67.7 (7.3)	31.8 (4.7)	22 (73.3)	30	ACR	30/0	III, IV	81.8 (30.5)	None	0, 4, 8, 16	VAS	0, 1, 2	1	3
	(Asia)	Gr 2: HA + EX	66.1 (9.1)	32.6 (2.5)	16 (53.3)	30		30/0		75.1 (33.6)			KOOS	0, 1, 2	1	3
		Gr 3: DxTP + EX	64.8 (5.8)	32.4 (4.1)	19 (63.3)	30		30/0		75.4 (30.1)				0, 4, 8	4	3
		Gr 4: PTA + EX	70.0 (6.3)	33.2 (3.9)	18 (60)	30		30/0		70.2 (24.5)						

To be continued.

Supplementary Table S2. Continued

Study	Country	Study arm	Age (years)	BMI (kg/m²)	Female	*N*	OA dignosis	Involved side	K-L grade	Disease duration (months)	Co-intervention	Measured time point (week)	Main outcome measures	Injection protocol		
(year)[Referenae]	(area)		Mean (SD or range)	Mean (SD or range)	n (%)			Unilateral /Bilateral		Mean (SD, range)				Injection time (protocol)	Time interval per injection (week)	No. of injections
Rezasoltani 2021	Iran (Asia)	Gr 1: BoNTA	77.7 (7.3)	31.3 (4.7)	18 (72)	25	ACR	30/0	III, IV	≥3	None	0, 4, 12, 24	VAS	0	0	1
		Gr 2: PT	63.0 (8.0)	29.2 (3.5)	20 (80)	25							KOOS			
Saccomanno 2016	Italy (Europe)	Gr 1: HA	62.8 (13.2)	27.2 (3.0)[b]	42 (79.2)	53	ACR	NR	I-III	32.0 (35.6)[b]	None	0, 8, 16, 28	WOMAC	0, 2, 4	2	3
		Gr 2: EX	61.2 (10.1)	27.4 (3.6)[b]	33 (64.7)	51				38.0 (51.9)[b]						
		Gr 3: HA + EX	61.4 (9.7)	28.7 (3.9)[b]	38 (71.7)	53				42.7 (45.9)[b]						
Sadat-Ali 2021	Saudi Arabia (Asia)	Gr 1: MSC	56.2 (6.6)	NR	24 (80)	30	ACR	NR	II-IV	≥12	None	0, 12, 52, 104	VAS	0	0	1
		Gr 2: PT	56.8 (5.8)		16 (53.3)	30							KSS			
Sert 2020	Turkey (Asia)	Gr 1: DxTP + EX	55.7 (6.6)	30.0 (4.6)	18 (85.7)	21	ACR	NR	II, III	307.2 (94.8)	acetaminophen	0, 6, 18	VAS; WOMAC	0, 3	3	2
		Gr 2: PLA + EX	54.4 (7.3)	32.3 (3.7)	20 (90.9)	22				295.2 (152.4)						
		Gr 3: EX	52.0 (6.1)	27.6 (4.0)	17 (89.5)	19				306.0 (135.6)						
Sezgin 2005	Turkey (Asia)	Gr 1: HA + EX	59.9 (9.8)	30.2 (4.3)	18 (81.8)	22	ACR	NR	II, III	41.7 (36.6)	None	0, 4	VAS; WOMAC	0, 1, 2	1	3
		Gr 2: PLA + EX	59.4 (10.2)	29.3 (4.3)	13 (68.4)	19				31.0 (37.6)			25-m walk			
Shrestha 2018	Nepal (Asia)	Gr 1: CS + PT	67.4 (5.4)		35 (61.4)	57	ACR	NR	NR	NR	Aceclofenac	0, 2, 6, 12	VAS; WOMAC	0	0	1
		Gr 2: PLA + PT	67.1 (5.2)		37 (61.7)	60							KOOS			
Sit 2020	China (Asia)	Gr 1: DxTP + EX	62.8 (5.8)	24.0 (3.4)	27 (71.1)	38	ACR	NR	I-IV	116.4 (117.4)	None	0, 16, 26, 52	VAS; WOMAC	0, 4, 8, 16	4	4
		Gr 2: PTA + EX	63.7 (5.2)	25.0 (3.3)	27 (71.1)	38				98.4 (68.4)			TUG; 40-m walk			
Soliman 2016	Egypt (Africa)	Gr 1: DxTP(1) + PT	51.1 (12.1)	NR	39 (75)	52	ACR	NR	I-IV	82.7 (108.4)	None	0, 2, 6, 12, 24	VAS	0, 4, 8, 12, 16	4	3–5
		Gr 2: DxTP(2) + PT	51.0 (10.5)		39 (75)	52				70.6 (107.9)			WOMAC	0, 4, 8, 12, 16	4	3–5
		Gr 3: PT	52.8 (11.1)		18 (75)	24				72.4 (104.5)						
Su 2019	China (Asia)	Gr 1: PRP	57.4 (45-66)	NR	39 (65)	60	Radiographic	60/0	I, II	37.0 (24-47)	None	0, 1, 3, 5	VAS	0, 1, 2, 3, 4	1	5
		Gr 2: ESWT	58.6 (47-68)		40 (66.7)	60		60/0		35.6 (25-45)			WOMAC			
		Gr 3: PRP + ESWT	59.7 (47-69)		41 (68.3)	60		60/0		37.6 (26-47)			Lequesne			
Subazwari 2020	Pakistan (Asia)	Gr 1: CS + PT	60.2 (7.6)	NR	15 (68.2)	22	Symptomatic	NR	NR	NR	None	0, 8	VAS	0	0	1
		Gr 2: PT	63.6 (7.1)		14 (63.6)	22							WOMAC			

To be continued.

Supplementary Table S2. Continued

Study	Country	Study arm	Age (years)	BMI (kg/m^2)	Female	*N*	OA dignosis	Involved side	K-L grade	Disease duration (months)	Co-intervention	Measured time point (week)	Main outcome measures	Injection protocol		
(year)[Referenae]	(area)		Mean (SD or range)	Mean (SD or range)	n (%)			Unilateral /Bilateral		Mean (SD, range)				Injection time (protocol)	Time interval per injection (week)	No. of injections
Taftain 2021	Iran (Asia)	Gr 1: HA + EX	62.0 (9.9)	30.5 (4.5)	37 (86)	43	ACR	NR	II-IV	>3	None	0, 4, 12, 24	VAS	0, 1, 2	1	3
		Gr 2: PLA + EX	65.7 (9.7)	28.9 (2.9)	28 (93.3)	30							KOOS			
		Gr 3: PTA	61.2 (8.4)	28.1 (3.0)	21 (77.8)	27										
Tucker 2021	USA (America)	Gr 1: PRP + EX	57.2 (3.9)	29.1 (2.1)	3 (27.3)	11	Radiographic	NR	II, III	NR	None	0, 2, 12, 24, 52	VAS	0	0	1
		Gr 2: PLA + EX	57.5 (1.8)	30.9 (1.5)	4 (66.7)	6							WOMAC			
Uslu Guvendi 2018	Turkey (Asia)	Gr 1: CS + EX	62.8 (1.7)	31.1 (1.1)	15 (88.2)	17	ACR	NR	III	NR	paracetamol	0, 8, 24	VAS	0	0	1
		Gr 2: 1-injection PRP + EX	62.3 (1.6)	31.4 (0.7)	18 (94.7)	19							WOMAC	0	0	1
		Gr 3: 3-injection PRP + EX	60.4 (1.7)	31.0 (1.0)	13 (92.9)	14							Lequesne	0, 1, 2	1	3

[a]Study sample mean; [b]Value is estimated.

ACR, American College of Rheumatology criteria; PLA, Placebo; BMI, body mass index; K-L grade, Kellgren and Lawrence grading system for classification of osteoarthritis; ACS, autologous conditioned serum; BoNTA, botulinum toxin type A; CS, corticosteroid; DxTP, dextrose prolotherapy; HA, hyaluronic acid; MSC, mesenchymal stem cell; OZ, ozone; PRP, platelet-rich plasma; PRGF, plasma rich in growth factor; SVF, stromal vascular fraction; VAS, visual analog scale; WOMAC, Western Ontario and McMaster Universities Osteoarthritis scale; KOOS, Knee injury and Osteoarthritis Outcome Scores; HSS, Hospital for Special Surgery; IKDC, International Knee Documentation Committee; ISK, Index of severity for OA of the knee; JKOM, Japanese Knee Osteoarthritis Measure; KSS, Knee Society Score; OKS, Oxford Knee scale; GS, gait speed; TUG, timed up and go; 6MWD, 6-minute walk distance; EX, exercise; PA, physical activity; PT, physical therapy; PTA, physical modality agent; ESWT, extracorporeal shock wave therapy; NR, not reported.

Supplementary Table S3. Summary of methodological quality based on the PEDro classification scale

Study author (year)	Overall[a]	1[b]	2[b]	3[b]	4[b]	5[b]	6[b]	7[b]	8[b]	9[b]	10[b]
Acosta-Olivo 2014	6	Y		Y				Y	Y	Y	Y
Akan 2018	7	Y	Y	Y				Y	Y	Y	Y
Altman 2009	8	Y		Y	Y		Y	Y	Y	Y	Y
Angoorani 2015	5	Y		Y				Y		Y	Y
Anz 2020	5	Y		Y				Y		Y	Y
Atamaz 2006	7	Y		Y	Y		Y	Y		Y	Y
Auerbach 2002	6	Y		Y				Y	Y	Y	Y
Babaei-Ghazani 2018	9	Y	Y	Y	Y		Y	Y	Y	Y	Y
Babaei-Ghazani 2019	9	Y	Y	Y	Y		Y	Y	Y	Y	Y
Bao 2018	8	Y		Y		Y	Y	Y	Y	Y	Y
Baranova 2018	6	Y		Y				Y	Y	Y	Y
Basar 2021	7	Y	Y	Y		Y	Y			Y	Y
Baygutalp 2021	6	Y		Y				Y	Y	Y	Y
Bayramoglu 2003	6	Y		Y				Y	Y	Y	Y
Centeno 2018	6	Y	Y	Y				Y		Y	Y
Chen 2013	6	Y		Y			Y	Y		Y	Y
Cole 2017	8	Y		Y	Y		Y	Y	Y	Y	Y
de Sire 2020	6	Y		Y			Y	Y		Y	Y
DeCaria 2012	9	Y		Y	Y	Y	Y	Y	Y	Y	Y
Delgado-Enciso 2018	8	Y	Y	Y			Y	Y	Y	Y	Y
Delgado-Enciso 2019	7	Y	Y	Y			Y	Y		Y	Y
Deyle 2020	8	Y	Y	Y			Y	Y	Y	Y	Y
Di Sante 2012	7	Y		Y			Y	Y	Y	Y	Y
Dumais 2012	7	Y	Y	Y				Y	Y	Y	Y
Elerian 2016	9	Y	Y	Y	Y		Y	Y	Y	Y	Y
Elgendy 2020	6	Y		Y				Y	Y	Y	Y
Elik 2020	8	Y		Y	Y	Y	Y	Y		Y	Y
Filardo 2015	9	Y	Y	Y	Y		Y	Y	Y	Y	Y
Forogh 2016	6	Y		Y	Y		Y			Y	Y
Freitag 2019	7	Y	Y	Y				Y	Y	Y	Y
Gaballa 2019	6	Y		Y				Y	Y	Y	Y
García-Triana 2021	7	Y	Y	Y				Y	Y	Y	Y
Garza 2020	9	Y	Y	Y	Y	Y	Y		Y	Y	Y
Ghai 2019	9	Y	Y	Y	Y		Y	Y	Y	Y	Y
Hawkins 2012	6¶	Y	Y	Y				Y		Y	Y
Henriksen 2015	10	Y	Y	Y	Y	Y	Y	Y	Y	Y	Y
Hermans 2019	8	Y	Y	Y			Y	Y	Y	Y	Y
Huang 2005	7	Y	Y	Y			Y	Y		Y	Y
Ip 2015	10	Y	Y	Y	Y	Y	Y	Y	Y	Y	Y
Isik 2020	6	Y		Y	Y		Y			Y	Y
Karatosun 2006	7	Y		Y			Y	Y	Y	Y	Y
Jhan 2022	7	Y		Y			Y	Y	Y	Y	Y
Kaszyński 2022	7	Y		Y			Y	Y	Y	Y	Y
Kawasaki 2009	7	Y		Y			Y	Y	Y	Y	Y
Khalifeh Soltani 2019	9	Y		Y	Y	Y	Y	Y	Y	Y	Y
Kon 2018	8	Y	Y	Y	Y		Y	Y		Y	Y

To be continued.

Supplementary Table S3. Continued

Study author (year)	Overall[a]	1[b]	2[b]	3[b]	4[b]	5[b]	6[b]	7[b]	8[b]	9[b]	10[b]
Kon 2020	8	Y	Y	Y	Y		Y	Y		Y	Y
Lee 2017	7	Y		Y			Y	Y	Y	Y	Y
Liu 2019	6	Y		Y				Y	Y	Y	Y
Lucangeli 2001	6	Y		Y				Y	Y	Y	Y
McAlindon 2018	9	Y	Y	Y	Y		Y	Y	Y	Y	Y
Nishida 2021	9	Y	Y	Y	Y		Y	Y	Y	Y	Y
Paker 2006	7	Y	Y	Y			Y	Y		Y	Y
Paolucci 2021	6	Y		Y			Y	Y		Y	Y
Parfitt 2006	5	Y		Y				Y		Y	Y
Paterson 2016	9	Y	Y	Y	Y	Y	Y	Y		Y	Y
Petrella 2002	8	Y		Y	Y	Y	Y	Y		Y	Y
Qamar 2021	8[¶]	Y		Y	Y		Y	Y	Y	Y	Y
Rabago 2013	9	Y	Y	Y	Y	Y	Y	Y		Y	Y
Raeissadat 2015	5	Y		Y				Y		Y	Y
Raeissadat 2018	8	Y	Y	Y	Y		Y	Y		Y	Y
Raeissadat 2020a	6	Y		Y			Y	Y		Y	Y
Raeissadat 2020b	8	Y	Y	Y			Y	Y	Y	Y	Y
Raeissadat 2021	8	Y	Y	Y	Y	Y	Y			Y	Y
Rayegani 2014	5	Y		Y				Y		Y	Y
Raynauld 2002	7	Y	Y	Y				Y	Y	Y	Y
Rezasoltani 2020	6	Y		Y				Y	Y	Y	Y
Rezasoltani 2021	6	Y		Y				Y	Y	Y	Y
Saccomanno 2016	7	Y	Y	Y			Y	Y		Y	Y
Sadat-Ali 2021	5	Y		Y				Y		Y	Y
Sert 2020	8	Y	Y	Y	Y		Y	Y		Y	Y
Sezgin 2005	8	Y		Y	Y		Y	Y	Y	Y	Y
Shrestha 2018	9	Y	Y	Y	Y		Y	Y	Y	Y	Y
Sit 2020	10	Y	Y	Y	Y	Y	Y	Y	Y	Y	Y
Soliman 2016	6[¶]	Y		Y				Y	Y	Y	Y
Su 2019	6	Y		Y				Y	Y	Y	Y
Subazwari 2020	6	Y		Y				Y	Y	Y	Y
Taftain 2021	7	Y	Y	Y			Y	Y		Y	Y
Tucker 2021	8	Y		Y	Y		Y	Y	Y	Y	Y
Uslu Guvendi 2018	6	Y		Y			Y	Y		Y	Y
Summary*		80	36	80	31	13	53	75	49	80	80

PEDro = Physiotherapy Evidence Database.
[¶]Score was determined by a third assessor.
*Calculated as the number of studies that satisfied the criteria.
[a]Points of methodological quality are denoted as "Y" for fulfilled criteria.
[b]PEDro classification scale: 1 = random allocation, 2 = concealed allocation, 3 = similarity at the baseline, 4 = subject blinding, 5 = therapist blinding, 6 = assessor blinding, 7 = over 85% follow-up for at least one key outcome, 8 = intention-to-treat analysis, 9 = between-group statistical comparison for at least one key outcome, 10 = point and variability measures for at least one key outcome.

Supplementary Table S4. League table for pairwise and network meta-analysis of mean change in pain score from baseline

	Direct evidence of pairwise meta-analyses (row compared with column)																	
	V1	V2	V3	V4	V5	V6	V7	V8	V9	V10	V11	V12	V13	V14	V15	V16	V17	V18
V1	ACS + PT	.	.	.	.	.	.	.	.	.	.	.	.	.	.	.	.	-0.66 (-2.05, 0.73)
V2	1.79 (-0.10, 3.67)	BoNTA + PT	.	-0.32 (-2.24, 1.60)	-3.94 (-5.38, -2.50)	.	.	.	.	.	.	.	.	.	.	.	-0.95 (-2.06, 0.17)	.
V3	0.34 (-1.37, 2.05)	-1.45 (-2.63, -0.26)	CS + PT	.	.	.	0.38 (-1.00, 1.76)	.	2.16 (0.74, 3.58)	.	-0.64 (-1.82, 0.54)	.	.	.	.	.	-1.31 (-2.14, -0.49)	.
V4	1.88 (0.14, 3.61)	0.09 (-1.07, 1.25)	1.54 (0.62, 2.46)	DxTP + PT	-3.18 (-5.14, -1.21)	.	0.37 (-1.56, 2.30)	.	.	.	.	-0.12 (-2.03, 1.80)	.	.	.	.	-2.14 (-2.90, -1.39)	.
V5	0.30 (-1.32, 1.92)	-1.48 (-2.53, -0.44)	-0.04 (-0.75, 0.67)	-1.57 (-2.35, -0.80)	HA + PT	.	-0.04 (-1.00, 0.92)	0.97 (-0.37, 2.31)	0.26 (-0.60, 1.12)	.	.	.	0.23 (-1.66, 2.12)	.	.	.	-0.53 (-1.02, -0.05)	-0.62 (-1.75, 0.51)
V6	0.89 (-0.95, 2.73)	-0.90 (-2.25, 0.45)	0.55 (-0.53, 1.62)	-0.99 (-2.12, 0.14)	0.59 (-0.38, 1.55)	MSC + PT	.	.	-0.07 (-1.43, 1.29)	.	.	.	.	-0.40 (-2.34, 1.54)	.	.	-1.02 (-2.19, 0.16)	.
V7	0.60 (-1.13, 2.32)	-1.19 (-2.38, 0.01)	0.26 (-0.55, 1.07)	-1.28 (-2.20, -0.36)	0.29 (-0.39, 0.98)	-0.29 (-1.40, 0.82)	OZ + PT	1.81 (-0.09, 3.71)	1.72 (-0.18, 3.62)	.	.	.	.	.	-1.11 (-3.06, 0.84)	.	-1.00 (-2.36, 0.35)	.
V8	1.48 (-0.54, 3.49)	-0.31 (-1.89, 1.27)	1.14 (-0.23, 2.50)	-0.40 (-1.81, 1.01)	1.17 (-0.04, 2.39)	0.59 (-0.93, 2.10)	0.88 (-0.43, 2.19)	PRGF + PT	-0.09 (-1.98, 1.80)	.	.	.	.	.	.	.	.	.
V9	0.80 (-0.86, 2.47)	-0.98 (-2.09, 0.13)	0.47 (-0.26, 1.19)	-1.07 (-1.90, -0.24)	0.50 (-0.04, 1.05)	-0.08 (-1.00, 0.84)	0.21 (-0.56, 0.98)	-0.67 (-1.94, 0.60)	PRP + PT	.	.	.	.	.	.	-0.75 (-2.64, 1.13)	-0.63 (-1.28, 0.02)	.
V10	0.96 (-1.57, 3.48)	-0.83 (-3.02, 1.37)	0.62 (-1.42, 2.66)	-0.92 (-2.98, 1.15)	0.66 (-1.33, 2.64)	0.07 (-2.07, 2.21)	0.36 (-1.70, 2.42)	-0.52 (-2.83, 1.79)	0.15 (-1.85, 2.16)	BoNTA	.	.	.	.	.	.	-1.16 (-3.11, 0.78)	.
V11	-0.46 (-2.24, 1.32)	-2.24 (-3.58, -0.91)	-0.80 (-1.71, 0.11)	-2.34 (-3.45, -1.22)	-0.76 (-1.72, 0.19)	-1.35 (-2.60, -0.09)	-1.06 (-2.13, 0.02)	-1.93 (-3.45, -0.42)	-1.26 (-2.25, -0.28)	-1.42 (-3.56, 0.72)	CS	.	.	.	.	.	0.69 (-0.43, 1.81)	-0.77 (-2.73, 1.18)
V12	0.70 (-1.28, 2.68)	-1.09 (-2.73, 0.55)	0.36 (-1.09, 1.81)	-1.18 (-2.51, 0.16)	0.39 (-0.96, 1.75)	-0.19 (-1.78, 1.40)	0.10 (-1.36, 1.56)	-0.78 (-2.58, 1.02)	-0.11 (-1.50, 1.29)	-0.26 (-2.61, 2.09)	1.16 (-0.41, 2.72)	DxTP	.	.	.	.	-0.27 (-2.20, 1.66)	-0.37 (-2.30, 1.56)
V13	-0.07 (-1.81, 1.67)	-1.86 (-3.07, -0.65)	-0.41 (-1.32, 0.51)	-1.95 (-2.91, -0.98)	-0.37 (-1.14, 0.39)	-0.96 (-2.08, 0.16)	-0.67 (-1.61, 0.28)	-1.55 (-2.96, -0.13)	-0.87 (-1.70, -0.05)	-1.03 (-3.09, 1.03)	0.39 (-0.72, 1.50)	-0.77 (-2.24, 0.71)	HA	.	.	.	-0.08 (-0.76, 0.61)	.
V14	0.64 (-1.43, 2.70)	-1.15 (-2.79, 0.49)	0.30 (-1.14, 1.73)	-1.24 (-2.71, 0.23)	0.33 (-1.02, 1.69)	-0.25 (-1.67, 1.17)	0.04 (-1.42, 1.50)	-0.84 (-2.63, 0.95)	-0.17 (-1.53, 1.19)	-0.32 (-2.66, 2.01)	1.10 (-0.47, 2.66)	-0.06 (-1.91, 1.79)	0.71 (-0.75, 2.16)	MSC	.	.	-0.80 (-2.17, 0.58)	.
V15	-0.35 (-2.44, 1.74)	-2.14 (-3.81, -0.46)	-0.69 (-2.14, 0.76)	-2.23 (-3.73, -0.73)	-0.65 (-2.03, 0.72)	-1.24 (-2.85, 0.37)	-0.95 (-2.31, 0.41)	-1.83 (-3.62, -0.03)	-1.16 (-2.56, 0.25)	-1.31 (-3.68, 1.06)	0.11 (-1.49, 1.70)	-1.05 (-2.92, 0.83)	-0.28 (-1.78, 1.22)	-0.99 (-2.85, 0.88)	OZ	0.81 (-1.15, 2.77)	-0.16 (-2.11, 1.80)	.
V16	0.40 (-1.51, 2.31)	-1.39 (-2.83, 0.06)	0.06 (-1.14, 1.26)	-1.48 (-2.72, -0.23)	0.10 (-1.01, 1.20)	-0.49 (-1.85, 0.87)	-0.20 (-1.40, 1.01)	-1.08 (-2.68, 0.53)	-0.41 (-1.51, 0.69)	-0.56 (-2.77, 1.65)	0.86 (-0.50, 2.22)	-0.30 (-1.97, 1.38)	0.47 (-0.77, 1.70)	-0.24 (-1.89, 1.42)	0.75 (-0.75, 2.25)	PRP	-0.63 (-1.74, 0.48)	.
V17	-0.20 (-1.81, 1.41)	-1.99 (-3.00, -0.98)	-0.54 (-1.17, 0.08)	-2.08 (-2.77, -1.39)	-0.51 (-0.91, -0.10)	-1.09 (-1.99, -0.19)	-0.80 (-1.48, -0.12)	-1.68 (-2.93, -0.43)	-1.01 (-1.49, -0.52)	-1.16 (-3.11, 0.78)	0.26 (-0.63, 1.14)	-0.90 (-2.22, 0.42)	-0.13 (-0.80, 0.54)	-0.84 (-2.13, 0.45)	0.15 (-1.20, 1.49)	-0.60 (-1.64, 0.44)	PT	-1.06 (-2.05, -0.08)
V18	-0.66 (-2.05, 0.73)	-2.45 (-3.73, -1.17)	-1.00 (-1.99, 0.00)	-2.54 (-3.57, -1.50)	-0.96 (-1.80, -0.13)	-1.55 (-2.75, -0.34)	-1.26 (-2.28, -0.23)	-2.14 (-3.59, -0.68)	-1.46 (-2.39, -0.54)	-1.62 (-3.73, 0.49)	-0.20 (-1.32, 0.92)	-1.36 (-2.77, 0.06)	-0.59 (-1.64, 0.46)	-1.30 (-2.82, 0.23)	-0.31 (-1.87, 1.25)	-1.06 (-2.37, 0.25)	-0.46 (-1.27, 0.36)	UC
	Relative effects of NMA (column compared with row)																	

Pairwise (upper right portion) and network (lower left portion) meta-analysis results are presented for mean change (from baseline) in pain outcomes. Effect estimation is presented in standardized mean difference (SMD) with 95% CI. Significant results are marked in red.

NMA, network meta-analysis; CS, corticosteroid; BoNTA, botulinum toxin type A; HA, hyaluronic acid: OZ, ozone; DxTP, dextrose prolotherapy; PRP, platelet-rich plasma; MSC, mesenchymal stem cell; PRGF, plasma rich in growth factor; ACS, autologous conditioned serum; PT, physical therapy; UC, usual care.

Supplementary Table S5. GRADE certainty ratings for each outcome

Treatment (Common comparator: UC)	Outcome measure (GRADE certainty of evidence)[a]		
	Pain	Global function	Walking capability
Combined therapy			
ACS + PT	⊕⊕⊖⊖[de]	⊕⊖⊖⊖[def]	
BoNTA + PT	⊕⊕⊕⊖[b]	⊕⊕⊖⊖[bf]	
CS + PT	⊕⊕⊖⊖[bd]	⊕⊕⊖⊖[bf]	⊕⊕⊖⊖[be]
DxTP + PT	⊕⊕⊕⊖[b]	⊕⊕⊖⊖[bf]	⊕⊕⊖⊖[be]
HA + PT	⊕⊕⊖⊖[bc]	⊕⊖⊖⊖[bcf]	⊕⊕⊕⊖[c]
MSC + PT	⊕⊕⊕⊖[b]	⊕⊕⊖⊖[bf]	⊕⊕⊕⊖[e]
OZ + PT	⊕⊕⊕⊖[b]	⊕⊕⊖⊖[bf]	⊕⊕⊕⊖[b]
PRGF + PT	⊕⊕⊕⊖[e]	⊕⊕⊖⊖[ef]	⊕⊕⊕⊖[e]
PRP + PT	⊕⊕⊕⊖[b]	⊕⊕⊖⊖[bf]	⊕⊕⊕⊖[b]
SVF + PT		⊕⊕⊖⊖[ef]	
Monotherapy			
BoNTA	⊕⊖⊖⊖[bde]	⊕⊖⊖⊖[bef]	
CS	⊕⊖⊖⊖[bcd]	⊕⊖⊖⊖[bcdf]	⊕⊕⊖⊖[be]
DxTP	⊕⊖⊖⊖[bcde]	⊕⊖⊖⊖[bcef]	
HA	⊕⊕⊖⊖[bd]	⊕⊕⊖⊖[bf]	⊕⊕⊖⊖[be]
MSC	⊕⊖⊖⊖[bde]	⊕⊖⊖⊖[bef]	
OZ	⊕⊖⊖⊖[bde]	⊕⊖⊖⊖[bef]	⊕⊖⊖⊖[bde]
PRP	⊕⊕⊖⊖[bd]	⊕⊕⊖⊖[bf]	⊕⊖⊖⊖[bde]
PT	⊕⊖⊖⊖[bcd]	⊕⊖⊖⊖[bcf]	⊕⊕⊖⊖[bc]

[a]Certainty of evidence is graded as follows:
High: ⊕⊕⊕⊕; Moderate: ⊕⊕⊕⊖; Low: ⊕⊕⊖⊖; Very low: ⊕⊖⊖⊖
[b]There is unclear or high risk of bias (i.e., PEDro score < 7) within the studies.
[c]The statistical heterogeneity is high.
[d]95% confidence interval is wide and imprecise.
[e]Insufficient number of studies (< 5) and sample size (< 30).
[f]Presumably publication bias.
GRADE, Grading of Recommendations, Assessment, Development and Evaluations; ACS, autologous conditioned serum; BoNTA, botulinum toxin type A; CS, corticosteroid; DxTP, dextrose prolotherapy; HA, hyaluronic acid; MSC, mesenchymal stem cell; OZ, ozone; PRP, platelet-rich plasma; PRGF, plasma rich in growth factor; PT, physical therapy; SVF, stromal vascular fraction; UC, usual care.

Supplementary Table S6. League table for pairwise and network meta-analysis of mean change in global function from baseline

	V1	V2	V3	V4	V5	V6	V7	V8	V9	V10	V11	V12	V13	V14	V15	V16	V17	V18	V19
	Direct evidence of pairwise meta-analyses (row compared with column)																		
V1	ACS + PT	.	.	.	.	.	.	.	.	.	.	.	.	.	.	.	.	.	0.17 (-0.88, 1.23)
V2	-1.94 (-3.37, -0.52)	BoNTA + PT	.	-0.10 (-1.53, 1.34)	1.04 (0.01, 2.07)	.	.	.	.	.	.	.	.	.	.	.	.	1.18 (0.33, 2.02)	.
V3	-0.79 (-2.10, 0.52)	1.15 (0.25, 2.05)	CS + PT	.	.	.	-0.08 (-1.12, 0.96)	.	-2.00 (-3.10, -0.89)	.	.	0.47 (-0.45, 1.39)	.	.	.	.	.	0.68 (-0.02, 1.39)	.
V4	-2.11 (-3.42, -0.80)	-0.17 (-1.03, 0.70)	-1.32 (-2.03, -0.61)	DxTP + PT	1.10 (-0.34, 2.55)	.	-0.01 (-1.46, 1.44)	.	.	.	.	.	-0.58 (-2.01, 0.86)	.	.	.	.	1.53 (0.97, 2.08)	.
V5	-1.19 (-2.42, 0.04)	0.76 (-0.02, 1.53)	-0.39 (-0.96, 0.17)	0.92 (0.35, 1.50)	HA + PT	.	0.16 (-0.56, 0.88)	-0.99 (-1.99, 0.00)	-0.53 (-1.17, 0.11)	.	.	.	.	0.02 (-1.38, 1.41)	.	.	.	0.47 (0.11, 0.83)	1.16 (0.31, 2.02)
V6	-1.85 (-3.25, -0.45)	0.09 (-0.93, 1.11)	-1.06 (-1.91, -0.22)	0.26 (-0.60, 1.11)	-0.67 (-1.41, 0.07)	MSC + PT	.	.	0.22 (-0.80, 1.24)	.	.	.	.	.	0.12 (-1.34, 1.58)	.	.	0.94 (0.02, 1.86)	.
V7	-1.06 (-2.37, 0.24)	0.88 (-0.01, 1.77)	-0.27 (-0.90, 0.35)	1.05 (0.36, 1.73)	0.12 (-0.39, 0.64)	0.79 (-0.05, 1.63)	OZ + PT	-1.33 (-2.73, 0.08)	-1.22 (-2.62, 0.19)	.	.	.	.	.	.	-0.31 (-1.77, 1.14)	.	0.58 (-0.43, 1.60)	.
V8	-2.16 (-3.67, -0.64)	-0.21 (-1.38, 0.96)	-1.36 (-2.39, -0.34)	-0.05 (-1.09, 1.00)	-0.97 (-1.87, -0.07)	-0.30 (-1.44, 0.83)	-1.09 (-2.07, -0.12)	PRGF + PT	0.11 (-1.29, 1.51)	.	.	.	.	.	.	.	.	.	.
V9	-1.77 (-3.04, -0.51)	0.17 (-0.65, 0.99)	-0.98 (-1.55, -0.41)	0.34 (-0.29, 0.96)	-0.59 (-1.00, -0.18)	0.08 (-0.62, 0.79)	-0.71 (-1.29, -0.13)	0.38 (-0.56, 1.33)	PRP + PT	.	.	.	.	.	.	.	0.21 (-1.18, 1.60)	0.80 (0.30, 1.30)	.
V10	-2.37 (-4.33, -0.40)	-0.43 (-2.14, 1.29)	-1.58 (-3.20, 0.05)	-0.26 (-1.88, 1.36)	-1.18 (-2.75, 0.39)	-0.51 (-2.20, 1.17)	-1.30 (-2.93, 0.32)	-0.21 (-2.01, 1.58)	-0.60 (-2.18, 0.99)	SVF + PT	.	.	.	.	.	.	.	1.60 (0.06, 3.14)	.
V11	-1.50 (-3.40, 0.40)	0.44 (-1.20, 2.08)	-0.71 (-2.25, 0.84)	0.61 (-0.94, 2.16)	-0.31 (-1.80, 1.18)	0.35 (-1.26, 1.97)	-0.44 (-1.98, 1.11)	0.66 (-1.07, 2.38)	0.27 (-1.23, 1.78)	0.87 (-1.25, 2.99)	BoNTA	.	.	.	.	.	.	0.74 (-0.72, 2.19)	.
V12	0.15 (-1.20, 1.50)	2.09 (1.08, 3.11)	0.94 (0.23, 1.65)	2.26 (1.41, 3.11)	1.34 (0.60, 2.07)	2.00 (1.04, 2.97)	1.21 (0.40, 2.03)	2.31 (1.17, 3.44)	1.92 (1.16, 2.68)	2.52 (0.83, 4.20)	1.65 (0.04, 3.26)	CS	.	.	.	.	.	-1.70 (-2.57, -0.82)	-0.16 (-1.64, 1.31)
V13	-1.66 (-3.16, -0.16)	0.28 (-0.94, 1.51)	-0.87 (-1.97, 0.24)	0.45 (-0.55, 1.45)	-0.47 (-1.49, 0.55)	0.20 (-1.01, 1.40)	-0.60 (-1.69, 0.50)	0.50 (-0.85, 1.84)	0.11 (-0.94, 1.16)	0.71 (-1.12, 2.54)	-0.16 (-1.92, 1.60)	-1.81 (-3.00, -0.62)	DxTP	.	.	.	.	0.52 (-0.93, 1.97)	0.62 (-0.83, 2.08)
V14	-1.03 (-2.35, 0.29)	0.91 (0.01, 1.81)	-0.24 (-0.96, 0.47)	1.08 (0.36, 1.79)	0.15 (-0.42, 0.73)	0.82 (-0.03, 1.68)	0.03 (-0.68, 0.74)	1.12 (0.07, 2.17)	0.74 (0.12, 1.36)	1.33 (-0.29, 2.95)	0.47 (-1.08, 2.01)	-1.18 (-2.03, -0.33)	0.63 (-0.48, 1.73)	HA	.	.	.	0.26 (-0.26, 0.77)	.
V15	-1.59 (-3.15, -0.03)	0.35 (-0.88, 1.58)	-0.80 (-1.90, 0.29)	0.52 (-0.58, 1.62)	-0.41 (-1.42, 0.61)	0.26 (-0.81, 1.34)	-0.53 (-1.62, 0.57)	0.56 (-0.77, 1.90)	0.18 (-0.84, 1.20)	0.78 (-1.05, 2.60)	-0.09 (-1.85, 1.66)	-1.74 (-2.93, -0.55)	0.07 (-1.32, 1.45)	-0.56 (-1.65, 0.54)	MSC	.	.	0.71 (-0.32, 1.74)	.
V16	-1.09 (-2.67, 0.49)	0.85 (-0.40, 2.11)	-0.30 (-1.41, 0.81)	1.02 (-0.11, 2.14)	0.09 (-0.94, 1.13)	0.76 (-0.46, 1.98)	-0.03 (-1.05, 0.99)	1.06 (-0.28, 2.41)	0.68 (-0.38, 1.74)	1.28 (-0.57, 3.12)	0.41 (-1.37, 2.19)	-1.24 (-2.45, -0.03)	0.57 (-0.84, 1.98)	-0.06 (-1.19, 1.07)	0.50 (-0.90, 1.90)	OZ	-0.68 (-2.16, 0.80)	0.09 (-1.39, 1.57)	.
V17	-1.57 (-3.02, -0.12)	0.37 (-0.71, 1.45)	-0.78 (-1.70, 0.14)	0.54 (-0.39, 1.47)	-0.38 (-1.21, 0.44)	0.28 (-0.75, 1.31)	-0.51 (-1.41, 0.40)	0.58 (-0.61, 1.78)	0.20 (-0.62, 1.03)	0.80 (-0.93, 2.52)	-0.07 (-1.73, 1.58)	-1.72 (-2.75, -0.69)	0.09 (-1.17, 1.35)	-0.54 (-1.46, 0.39)	0.02 (-1.22, 1.27)	-0.48 (-1.61, 0.65)	PRP	0.65 (-0.19, 1.48)	.
V18	-0.76 (-1.99, 0.46)	1.18 (0.43, 1.93)	0.03 (-0.48, 0.54)	1.34 (0.83, 1.86)	0.42 (0.12, 0.72)	1.09 (0.40, 1.78)	0.30 (-0.21, 0.81)	1.39 (0.47, 2.32)	1.01 (0.64, 1.38)	1.60 (0.06, 3.14)	0.74 (-0.72, 2.19)	-0.91 (-1.60, -0.23)	0.89 (-0.09, 1.88)	0.27 (-0.23, 0.77)	0.83 (-0.15, 1.80)	0.33 (-0.69, 1.34)	0.81 (0.03, 1.59)	PT	1.70 (0.94, 2.46)
V19	0.17 (-0.88, 1.23)	2.12 (1.16, 3.07)	0.97 (0.19, 1.74)	2.28 (1.50, 3.06)	1.36 (0.73, 1.99)	2.03 (1.11, 2.95)	1.24 (0.46, 2.01)	2.33 (1.24, 3.42)	1.95 (1.24, 2.65)	2.54 (0.88, 4.20)	1.67 (0.09, 3.26)	0.02 (-0.83, 0.87)	1.83 (0.77, 2.90)	1.21 (0.41, 2.00)	1.77 (0.61, 2.92)	1.27 (0.09, 2.45)	1.74 (0.75, 2.74)	0.94 (0.32, 1.56)	UC
	Relative effects of NMA (column compared with row)																		

Pairwise (upper right portion) and network (lower left portion) meta-analysis results are presented for mean change (from baseline) in global function. Effect estimation is presented in standardized mean difference (SMD) with 95% CI. Significant results are marked in red.

NMA, network meta-analysis; CS, corticosteroid; BoNTA, botulinum toxin type A; HA, hyaluronic acid: OZ, ozone; DxTP, dextrose prolotherapy; PRP, platelet-rich plasma; MSC, mesenchymal stem cell; PRGF, plasma rich in growth factor; ACS, autologous conditioned serum; SVF, stromal vascular fraction of adipose tissue; PT, physical therapy; UC, usual care.

Supplementary Table S7. League table for pairwise and network meta-analysis of mean change in walking capability from baseline

	Direct evidence of pairwise meta-analyses (row compared with column)												
	**V1**	**V2**	**V3**	**V4**	**V5**	**V6**	**V7**	**V8**	**V9**	**V10**	**V11**	**V12**	**V13**
**V1**	CS + PT	.	.	.	.	.	1.21 (-0.17, 2.60)	0.03 (-1.63, 1.69)	.	.	.	0.14 (-1.12, 1.41)	.
**V2**	0.46 (-0.72, 1.63)	DxTP + PT	.	.	-0.40 (-1.73, 0.92)	.	.	.	.	.	.	0.14 (-0.63, 0.90)	.
**V3**	0.17 (-0.85, 1.19)	-0.29 (-1.18, 0.61)	HA + PT	.	0.16 (-1.10, 1.43)	-0.10 (-0.99, 0.80)	-0.15 (-1.41, 1.12)	.	.	.	.	0.61 (0.01, 1.21)	1.81 (0.86, 2.76)
**V4**	-0.51 (-2.24, 1.21)	-0.97 (-2.79, 0.85)	-0.69 (-2.37, 1.00)	MSC + PT	.	.	0.96 (-0.41, 2.33)	.	.	.	.	.	.
**V5**	0.27 (-0.92, 1.45)	-0.19 (-1.20, 0.81)	0.10 (-0.79, 0.98)	0.78 (-0.96, 2.52)	OZ + PT	-0.23 (-1.49, 1.04)	-0.31 (-1.57, 0.96)	.	.	.	.	0.61 (-0.72, 1.93)	.
**V6**	0.15 (-1.08, 1.38)	-0.31 (-1.47, 0.85)	-0.02 (-0.88, 0.83)	0.67 (-1.09, 2.42)	-0.12 (-1.16, 0.93)	PRGF + PT	-0.08 (-1.34, 1.18)	.	.	.	.	.	.
**V7**	0.44 (-0.61, 1.50)	-0.02 (-1.21, 1.18)	0.27 (-0.71, 1.25)	0.96 (-0.41, 2.33)	0.18 (-0.90, 1.26)	0.29 (-0.80, 1.38)	PRP + PT	.	.	.	.	.	.
**V8**	0.45 (-0.71, 1.61)	-0.01 (-1.29, 1.27)	0.28 (-0.88, 1.44)	0.97 (-0.96, 2.89)	0.18 (-1.15, 1.52)	0.30 (-1.08, 1.68)	0.01 (-1.34, 1.36)	CS	.	.	.	-0.15 (-1.39, 1.09)	.
**V9**	0.58 (-0.73, 1.88)	0.12 (-1.06, 1.30)	0.41 (-0.66, 1.47)	1.09 (-0.83, 3.02)	0.31 (-0.95, 1.57)	0.43 (-0.89, 1.75)	0.13 (-1.22, 1.49)	0.13 (-1.27, 1.52)	HA	.	.	-0.04 (-0.95, 0.87)	.
**V10**	0.53 (-1.11, 2.17)	0.07 (-1.47, 1.62)	0.36 (-1.10, 1.82)	1.04 (-1.12, 3.21)	0.26 (-1.35, 1.87)	0.38 (-1.28, 2.03)	0.09 (-1.59, 1.77)	0.08 (-1.63, 1.79)	-0.05 (-1.68, 1.58)	OZ	-0.07 (-1.42, 1.28)	0.01 (-1.35, 1.36)	.
**V11**	0.46 (-1.18, 2.10)	0.00 (-1.54, 1.55)	0.29 (-1.17, 1.75)	0.97 (-1.19, 3.14)	0.19 (-1.42, 1.80)	0.31 (-1.35, 1.96)	0.02 (-1.66, 1.70)	0.01 (-1.70, 1.72)	-0.12 (-1.75, 1.51)	-0.07 (-1.42, 1.28)	PRP	0.08 (-1.28, 1.43)	.
**V12**	0.54 (-0.39, 1.47)	0.08 (-0.67, 0.82)	0.37 (-0.18, 0.91)	1.05 (-0.64, 2.74)	0.27 (-0.60, 1.14)	0.39 (-0.57, 1.34)	0.09 (-0.90, 1.09)	0.09 (-0.96, 1.13)	-0.04 (-0.95, 0.87)	0.01 (-1.35, 1.36)	0.08 (-1.28, 1.43)	PT	2.49 (1.17, 3.82)
**V13**	2.03 (0.72, 3.34)	1.57 (0.37, 2.77)	1.86 (0.96, 2.76)	2.54 (0.66, 4.43)	1.76 (0.54, 2.98)	1.88 (0.65, 3.10)	1.59 (0.29, 2.88)	1.58 (0.16, 2.99)	1.45 (0.13, 2.78)	1.50 (-0.16, 3.16)	1.57 (-0.09, 3.23)	1.49 (0.53, 2.45)	UC
	Relative effects of NMA (column compared with row)												

Pairwise (upper right portion) and network (lower left portion) meta-analysis results are presented for mean change (from baseline) in walk capability. Effect estimation is presented in standardized mean difference (SMD) with 95% CI. Significant results are marked in red. NMA, network meta-analysis; CS, corticosteroid; BoNTA, botulinum toxin type A; HA, hyaluronic acid: OZ, ozone; DxTP, dextrose prolotherapy; PRP, platelet-rich plasma; MSC, mesenchymal stem cell; PRGF, plasma rich in growth factor; ACS, autologous conditioned serum; SVF, stromal vascular fraction of adipose tissue; PT, physical therapy; UC, usual care.

Supplementary Table S8. Associations of moderators with treatment efficiency for all outcome measures

	Knee pain[a]					Global function[a]					Walking capability[a]				
Moderator	N	B	SE	Median	95% CrI	N	B	SE	Median	95% CrI	N	B	SE	Median	95% CrI
Participant factor															
Age	77	0.343	0.0358	−0.857	−2.114, 20.715	77	−0.501	0.0059	−0.485	−2.167, 1.102	19	0.709	0.0691	0.084	−22.555, 24.129
BMI	56	−0.332	0.0055	−0.267	−1.015, 0.396	55	−6.269	0.1570	−3.139	−59.694, 38.515	16	−1.191	0.0589	−0.057	−26.417, 8.203
Sex distribution[b]	72	2.876	0.0712	−0.193	−13.755, 31.331	71	0.132	0.0055	0.132	−1.398, 1.665	**18**	**−8.147**	**0.0169**	**−8.245**	**−12.558, −2.883**
Area of population[c]	78	−0.022	0.0163	−0.434	−1.496, 7.192	**78**	**1.684**	**0.0049**	**1.677**	**0.327, 3.092**	19	1.446	0.0758	0.119	−16.223, 38.204
Disease duration	32	0.036	0.0061	0.047	−1.675, 1.756	32	−2.397	0.0119	−2.373	−5.798, 0.878	12	−1.285	0.0328	−1.031	−11.207, 7.733
KL III-IV proportion[d]	**62**	**−2.520**	**0.0335**	**−1.405**	**−23.157, −0.384**	61	0.716	0.0041	0.719	−0.419 1.840	**17**	**2.157**	**0.0038**	**2.162**	**1.045, 3.230**
Study design factor															
PEDro score	78	2.914	0.0655	−0.101	−1.484, 34.538	78	−0.103	0.0089	−0.071	−2.657, 2.295	19	0.527	0.065	0.138	−19.562, 21.040
Follow-up duration	78	−2.810	0.0591	−2.315	−31.072, 19.314	78	−0.198	0.0055	−0.014	−1.530, 1.503	19	0.118	0.035	0.180	−11.973, 9.970
Intervention factor															
PT treatment type[e]	78	0.255	0.0098	0.238	−2.309, 3.229	78	−0.029	0.0077	−0.037	−1.384, 1.363	**19**	**4.839**	**0.0088**	**4.884**	**2.169, 7.233**
Treatment composition[f]	78	2.344	0.0804	−0.501	−18.905, 31.636	78	−0.388	0.0058	−0.384	−1.987, 1.193	19	0.542	0.0062	0.521	−1.137, 2.344
Treatment duration	78	0.833	0.0047	0.802	−0.364, 2.210	78	0.226	0.0047	0.228	−1.106, 1.509	19	0.347	0.0348	0.091	−9.015, 11.913

[a]Data represents the change in effects associated with the moderator indicated. B, beta coefficient; SE, standard error; 95% CI, 95% credibility interval.

[b]The proportion of female participants in sample

[c]Code for regression model: America = 1; Europe = 2; Asian = 3; Africa = 4; Oceania = 5.

[d]The proportion of participants who had Kellgren and Lawrence grade ≥ III in sample

[e]Code for regression model: no physical agent modality or exercise = 1; physical agent modality = 2; exercise or physical activity = 3; mixed components = 4.

[f]Code for regression model: monotherapy = 1; combined treatment = 2.

95% CrI, credible interval; BMI, body mass index; KL, Kellgren and Lawrence grading system for classification of osteoarthritis; PEDro, Physiotherapy Evidence Database; EFD, energy flux density.

Supplementary Table S9. Summary of compliance & adverse events

Study (year)	Study arm	Group sample (n)	Withdraw, attrition rate, or drop out (number of patients)			Side effects and complications (number of patients)			serious adverse event (number of patients)		
			Related to treatment	Unrelated to treatment	Total sum	Related to treatment	Unrelated to treatment	Total sum	Related to treatment	Unrelated to treatment	Total sum
Acosta-Olivo 2014	Gr 1: PRP + EX	21	0	0	0	NR	NR		NR	NR	
	Gr 2: EX	21	0	0	0	NR	NR		NR	NR	
Akan 2018	Gr 1: PRP + EX	31	1	0	1	13	0	13	0	0	0
	Gr 2: EX	31	0	1	1	0	0	0	0	0	0
Altman 2009	Gr 1: HA + PT	293	11	23	34	41	45	148	0	9	9
	Gr 2: PLA + PT	295	6	28	34	31	36	159	0	10	10
Angoorani 2015	Gr 1: PRP	27	0	1	1	3	0	3	0	0	0
	Gr 2: PT	27	0	3	3	1	0	1	0	0	0
Anz 2020	Gr 1: MSC+PT	49	2	2	4	NR	NR		NR	NR	
	Gr 2: PRP+PT	41	1	1	2	NR	NR		NR	NR	
Atamaz 2006	Gr 1: HA	40	0	0	0	4	0	4	0	0	0
	Gr 2: HA	42	2	0	2	0	0	0	0	0	0
Auerbach 2002	Gr 3: PTA	56	3	0	3	NR	NR		NR	NR	
	Gr 1: HA+EX	53	1	1	2	NR	NR		NR	NR	
Babaei-Ghazani 2018	Gr 2: OZ+EX	31	0	0	0	0	0	0	0	0	0
	Gr 1: CS+EX	31	0	0	0	0	0	0	0	0	0
Babaei-Ghazani 2019	Gr 2: OZ + EX	16	0	0	0	NR	NR		NR	NR	
	Gr 1: CS + EX	16	0	0	0	NR	NR		NR	NR	
Bao 2018	Gr 2: OZ + EX	20	0	0	0	0	0	0	0	0	0
	Gr 1: HA + EX	20	0	0	0	0	0	0	0	0	0
	Gr 2: BoNTA + EX	20	0	0	0	0	0	0	0	0	0
Baranova 2018	Gr 3: PLA + EX	45	0	0	0	0	0	0	0	0	0
	Gr 1: OZ + PTA	44	0	0	0	0	0	0	0	0	0
Basar 2021	Gr 1: HA + PT	48	0	17	17	NR	NR		NR	NR	
	Gr 2: PT	48	0	3	3	NR	NR		NR	NR	
Baygutalp 2021	Gr 1: DxTP + EX	25	0	0	0	NR	NR		NR	NR	
	Gr 2: OZ + EX	25	0	0	0	NR	NR		NR	NR	
	Gr 3: EX	25	0	0	0	NR	NR		NR	NR	
Bayramoglu 2003	Gr 1: HA	16	0	0	0	0	0	0	0	0	0
	Gr 2: HA	12	0	0	0	0	0	0	0	0	0
	Gr 3: PT	9	0	0	0	0	0	0	0	0	0
Centeno 2018	Gr 1: MSC + EX	22	2	0	2	8	1	9	0	0	0
	Gr 2: MSC	26	2	2	4	8	1	9	0	0	0
	Gr 3: EX	22	1	0	1	0	0	0	0	0	0
Chen 2013	Gr 1: HA	27	0	0	0	2	0	2	0	0	0
	Gr 2: PTA	27	0	4	4	0	0	0	0	0	0
Cole 2017	Gr 1: HA + EX	59	3	6	9	NR	NR		NR	NR	
	Gr 2: PRP + EX	52	0	3	3	NR	NR		NR	NR	

To be continued.

Supplementary Table S9. Continued

Study (year)	Study arm	Group sample (n)	Withdraw, attrition rate, or drop out (number of patients)			Side effects and complications (number of patients)			serious adverse event (number of patients)		
			Related to treatment	Unrelated to treatment	Total sum	Related to treatment	Unrelated to treatment	Total sum	Related to treatment	Unrelated to treatment	Total sum
de Sire 2020	Gr 1: HA + EX	20	0	1	1	4	0	4	0	0	0
	Gr 2: OZ + EX	22	0	2	2	3	0	3	0	0	0
DeCaria 2012	Gr 1: HA + EX	15	15	0	0	0	0	0	0	0	0
	Gr 2: PLA + EX	15	15	0	0	0	0	0	0	0	0
Delgado-Enciso 2018	Gr 1: CS + PT	8	8	0	0	8	0	8	0	0	0
	Gr 2: PT	8	8	0	0	6	0	6	0	0	0
Delgado-Enciso 2019	Gr 1: CS + PT	119	1	11	12	115	0	115	0	0	0
	Gr 2: PT	118	7	6	13	78	0	78	0	0	0
Deyle 2020	Gr 1: CS	78	4	1	5	1	24	25	0	0	0
	Gr 2: PT	78	0	1	1	0	11	11	0	0	0
Di Sante 2012	Gr 1: CS	20	0	0	0	0	0	0	0	0	0
	Gr 2: PTA	20	0	0	0	0	0	0	0	0	0
	Gr 3: CS + PTA	20	0	0	0	0	0	0	0	0	0
Dumais 2012	Gr 1: DxTP + EX	54	2	2	4	1	0	1	0	0	0
	Gr 2: EX	54	0	3	3	0	0	0	0	0	0
Elerian 2016	Gr 1: CS	20	0	0	0	NR	NR		NR	NR	
	Gr 2: PTA(ESWT)	20	0	0	0	NR	NR		NR	NR	
	Gr 3: UC	20	0	0	0	NR	NR		NR	NR	
Elgendy 2020	Gr 1: PRP + PT	15	0	0	0	NR	NR		NR	NR	
	Gr 2: PT	30	0	0	0	NR	NR		NR	NR	
Elik 2020	Gr 1: PRP + EX	30	0	0	0	5	0	5	0	0	0
	Gr 2: PLA + EX	30	0	3	3	3	0	3	0	0	0
Filardo 2015	Gr 1: HA + PA	96	2	5	7	2	0	2	0	0	0
	Gr 2: PRP + PA	96	2	0	2	2	0	2	0	0	0
Forogh 2016	Gr 1: CS + EX	24	6	2	8	0	0	0	0	0	0
	Gr 2: PRP + EX	24	1	0	1	1	0	1	0	0	0
Freitag 2019	Gr 1: 1-injection MSC + EX	10	0	0	0	8	0	8	0	0	0
	Gr 2: 2-injection MSC + EX	10	0	0	0	9	0	9	0	0	0
	Gr 3: EX	10	0	0	0	0	0	0	0	0	0
Gaballa 2019	Gr 1: PRP	20	0	0	0	NR	0		0	0	0
	Gr 2: OZ	20	0	0	0	NR	0		0	0	0
	Gr 3: EX + PTA	20	0	0	0	NR	NR		0	0	0
García-Triana 2021	Gr 1: DxTP + PT	25	0	0	0	0	0	0	0	0	0
	Gr 2: PT	25	0	0	0	0	0	0	0	0	0
Garza 2020	Gr1: High-dose SVF + PA	13	3	0	3	3	0	3	0	0	0
	Gr2: Low-dose SVF + PA	13	3	0	3	0	0	0	0	0	0
	Gr3: PLA + PA	13	4	3	7	0	0	0	0	0	0
Ghai 2019	Gr 1: PRP + EX	20	0	0	0	1	0	1	0	0	0
	Gr 2: PLA + EX	20	0	0	0	0	0	0	0	0	0
Hawkins 2012	Gr 1: CS	15	0	1	1	NR	NR		NR	NR	
	Gr 2: CS + EX	17	0	3	3	NR	NR		NR	NR	

To be continued.

Supplementary Table S9. Continued

Study (year)	Study arm	Group sample (n)	Withdraw, attrition rate, or drop out (number of patients)			Side effects and complications (number of patients)			serious adverse event (number of patients)		
			Related to treatment	Unrelated to treatment	Total sum	Related to treatment	Unrelated to treatment	Total sum	Related to treatment	Unrelated to treatment	Total sum
Henriksen 2015	Gr 1: CS + EX	50	1	4	5	1	0	1	0	0	0
	Gr 2: PLA + EX	50	3	3	6	3	0	3	0	0	0
	Gr 3: PLA + PT	50	0	0	0	NR	NR		NR	NR	
Hermans 2019	Gr 1: HA + PT	77	1	1	2	40	7	47	0	0	0
	Gr 2: PT	79	0	4	4	23	6	29	0	0	0
Huang 2005	Gr 1: EX	35	5	0	5	5	0	5	0	0	0
	Gr 2: EX + PTA	35	3	0	3	3	0	3	0	0	0
	Gr 3: HA + EX + PTA	35	1	1	2	1	0	1	0	0	0
	Gr 4: UC	35	3	1	4	0	0	0	0	0	0
Ip 2015	Gr 1: HA + PT	70	0	0	0	1	0	1	0	0	0
	Gr 2: PLA + PT	70	0	0	0	0	0	0	0	0	0
Isik 2020	Gr 1: DxTP + PTA	37	4	2	6	4	0	4	0	0	0
	Gr 2: DxTP	39	5	2	7	5	0	5	0	0	0
Jhan 2022	Gr 1: HA + PT	15	0	0	0	NR	NR		NR	NR	
	Gr 2: PT	15	0	0	0	NR	NR		NR	NR	
	Gr 3: UC	15	0	0	0	NR	NR		NR	NR	
Kaszyński 2022	Gr 1: MSC + EX	20	0	0	0	NR	NR		NR	NR	
	Gr 2: PRP + EX	20	0	0	0	NR	NR		NR	NR	
Karatosun 2006	Gr 1: HA	52	14	7	21	14	0	14	0	0	0
	Gr 2: EX	53	0	0	0	0	0	0	0	0	0
Kawasaki 2009	Gr 1: HA	50	8	10	18	0	0	0	0	0	0
	Gr 2: EX	52	5	5	10	0	0	0	0	0	0
Khalifeh Soltani 2019	Gr 1: MSC + PA	10	0	0	0	4	0	4	0	0	0
	Gr 2: PLA + PA	10	0	0	0	0	0	0	0	0	0
Kon 2018	Gr 1: ACS + PA	31	0	2	2	14	0	14	0	0	0
	Gr 2: PLA + PA	15	0	1	1	6	0	6	0	0	0
Kon 2020	Gr 1: ACS + PA	14	0	0	0	0	0	0	0	0	0
	Gr 2: PA	26	0	1	1	0	0	0	0	0	0
Lee 2017	Gr 1: HA + PA	30	0	0	0	X (NR)	0		0	0	0
	Gr 2: ESWT + PA	31	0	0	0	X (NR)	0		0	0	0
Liu 2019	Gr 1: HA	39	0	0	0	0	0	0	0	0	0
	Gr 2: PTA	38	0	0	0	0	0	0	0	0	0
Lucangeli 2001	Gr 1: HA + PT	10	0	0	0	0	0	0	0	0	0
	Gr 2: PT	10	0	0	0	0	0	0	0	0	0
McAlindon 2018	Gr 1: BoNTA (high dose) + PT	44	2	3	5	2	22	24	0	0	0
	Gr 2: BoNTA (low dose) + PT	43	3	3	6	1	29	30	0	0	0
	Gr 3: PLA + PT	89	2	5	7	3	48	51	0	0	0
Nishida 2021	Gr 1: HA + PT	88	4	2	6	5	45	50	0	1	1
	Gr 2: PLA + PT	89	6	2	8	5	47	52	0	2	2
Paker 2006	Gr 1: HA	30	2	3	5	0	0	0	0	0	0
	Gr 2: PTA	30	2	1	3	1	0	1	0	0	0
Paolucci 2021	Gr 1: OZ + PTA + EX	29	4	0	4	0	0	0	0	0	0
	Gr 2: OZ	26	2	0	2	0	0	0	0	0	0
Parfitt 2006	Gr 1: CS + EX	8	1	0	1	NR	NR		NR	NR	
	Gr 2: CS	5	0	0	0	NR	NR		NR	NR	
	Gr 3: PT	24	0	0	0	NR	NR		NR	NR	

To be continued.

Supplementary Table S9. Continued

Study (year)	Study arm	Group sample (n)	Withdraw, attrition rate, or drop out (number of patients)			Side effects and complications (number of patients)			serious adverse event (number of patients)		
			Related to treatment	Unrelated to treatment	Total sum	Related to treatment	Unrelated to treatment	Total sum	Related to treatment	Unrelated to treatment	Total sum
Paterson 2016	Gr 1: HA + PA	11	1	1	2	0	0	0	0	0	0
	Gr 2: PRP + PA	12	1	1	2	2	0	2	0	0	0
Petrella 2002	Gr 1: HA + EX + NSAID	30	0	1	1	X (NR)	X (NR)		0	0	0
	Gr 2: EX + NSAID	30	0	4	4	X (NR)	X (NR)		0	0	0
	Gr 3: HA + EX	30	0	5	5	X (NR)	X (NR)		0	0	0
	Gr 4: EX	30	0	2	2	X (NR)	X (NR)		0	0	0
Qamar 2021	Gr 1: PRP + PT	50	0	0	0	NR	NR		NR	NR	
	Gr 2: PLA + PT	50	0	0	0	NR	NR		NR	NR	
Rabago 2013	Gr 1: DxTP	33	0	3	3	30	0	30	0	0	0
	Gr 2: EX	34	0	12	12	31	0	31	0	0	0
Raeissadat 2015	Gr 1: HA + EX	73	9	2	11	NR	NR		NR	NR	
	Gr 2: PRP + EX	87	8	2	10	NR	NR		NR	NR	
Raeissadat 2018	Gr 1: HA + EX	87	3	10	13	0	0	0	0	0	0
	Gr 2: OZ + EX	87	5	15	20	0	0	0	0	0	0
Raeissadat 2020a	Gr 1: PRP + EX	23	0	2	2	NR	NR		NR	NR	
	Gr 2: EX	23	0	2	2	NR	NR		NR	NR	
Raeissadat 2020b	Gr 1: HA + EX	59	7	0	7	3	0	3	0	0	0
	Gr 2: PRGF + EX	60	7	3	10	10	0	10	0	0	0
Raeissadat 2021	Gr 1: HA + EX	59	3	7	10	12	0	12	0	0	0
	Gr 2: PRP + EX	59	4	3	7	17	0	17	0	0	0
	Gr 3: PRGF + EX	60	3	6	9	21	0	21	0	0	0
	Gr 4: OZ + EX	60	3	9	12	21	0	21	0	0	0
Rayegani 2014	Gr 1: PRP + EX	32	0	1	1	NR	NR		NR	NR	
	Gr 2: EX	33	0	2	2	NR	NR		NR	NR	
Raynauld 2002	Gr 1: HA + PT	127	3	0	3	119	0	119	0	0	0
	Gr 2: PT	128	21	0	21	96	0	96	0	1	1
Rezasoltani 2020	Gr 1: BoNTA + EX	30	1	0	1	NR	NR		NR	NR	
	Gr 2: HA + EX	30	2	1	3	NR	NR		NR	NR	
	Gr 3: DxTP + EX	30	1	1	2	NR	NR		NR	NR	
	Gr 4: PTA + EX	30	0	2	2	NR	NR		NR	NR	
Rezasoltani 2021	Gr 1: BoNTA	25	0	0	0	2	0	2	0	0	0
	Gr 2: PT	25	0	0	0	0	0	0	0	0	0
Saccomanno 2016	Gr 1: HA	55	0	11	11	0	0	0	0	0	0
	Gr 2: EX	55	2	12	14	0	0	0	0	0	0
	Gr 3: HA + EX	55	0	11	11	0	0	0	0	0	0
Sadat-Ali 2021	Gr 1: MSC	30	0	0	0	0	0	0	0	0	0
	Gr 2: PT	30	3	0	3	1	0	1	0	0	0
Sert 2020	Gr 1: DxTP + EX	22	0	1	1	NR	NR		NR	NR	
	Gr 2: PLA + EX	22	0	0	0	NR	NR		NR	NR	
	Gr 3: EX	22	0	3	3	NR	NR		NR	NR	

To be continued.

Supplementary Table S9. Continued

Study (year)	Study arm	Group sample (n)	Withdraw, attrition rate, or drop out (number of patients)			Side effects and complications (number of patients)			serious adverse event (number of patients)		
			Related to treatment	Unrelated to treatment	Total sum	Related to treatment	Unrelated to treatment	Total sum	Related to treatment	Unrelated to treatment	Total sum
Sezgin 2005	Gr 1: HA + EX	22	0	0	0	0	0	0	0	0	0
	Gr 2: PLA + EX	19	0	0	0	0	0	0	0	0	0
Shrestha 2018	Gr 1: CS + PT	85	0	28	28	1	0	1	0	0	0
	Gr 2: PLA + PT	86	0	26	26	0	0	0	0	0	0
Sit 2020	Gr 1: DxTP + EX	38	1	0	1	0	0	0	0	2	2
	Gr 2: PTA + EX	38	1	2	3	0	0	0	0	6	6
Soliman 2016	Gr 1: DxTP(1) + PT	52	0	0	0	NR	NR		NR	NR	
	Gr 2: DxTP(2) + PT	52	0	0	0	NR	NR		NR	NR	
	Gr 3: PT	24	0	0	0	NR	NR		NR	NR	
Su 2019	Gr 1: PRP	60	0	0	0	NR	NR		NR	NR	
	Gr 2: ESWT	60	0	0	0	NR	NR		NR	NR	
	Gr 3: PRP + ESWT	60	0	0	0	NR	NR		NR	NR	
Subazwari 2020	Gr 1: CS + PT	22	0	0	0	NR	NR		NR	NR	
	Gr 2: PT	22	0	0	0	NR	NR		NR	NR	
Taftain 2021	Gr 1: HA + EX	44	0	1	1	2	0	2	0	0	0
	Gr 2: PLA + EX	43	0	13	13	2	0	2	0	0	0
	Gr 3: PTA	43	3	13	16	0	0	0	0	0	0
Tucker 2021	Gr 1: PRP + EX	11	0	1	1	1	0	1	0	0	0
	Gr 2: PLA + EX	6	0	0	0	0	0	0	0	0	0
Uslu Guvendi 2018	Gr 1: CS + EX	19	0	2	2	NR	NR		NR	NR	
	Gr 2: 1-injection PRP + EX	19	0	0	0	NR	NR		NR	NR	
	Gr 3: 3-injection PRP + EX	19	5	0	5	NR	NR		NR	NR	

PLA, Placebo; ACS, autologous conditioned serum; BoNTA, botulinum toxin type A; CS, corticosteroid; DxTP, dextrose prolotherapy; HA, hyaluronic acid; MSC, mesenchymal stem cell; OZ, ozone; PRP, platelet-rich plasma; PRGF, plasma rich in growth factor; SVF, stromal vascular fraction; EX, exercise; PA, physical activity; PT, physical therapy; PTA, physical modality agent; ESWT, extracorporeal shock wave therapy; UC, usual care; NR, not reported.

Supplementary Figure S1. Forest plot of node-splitting results for pain

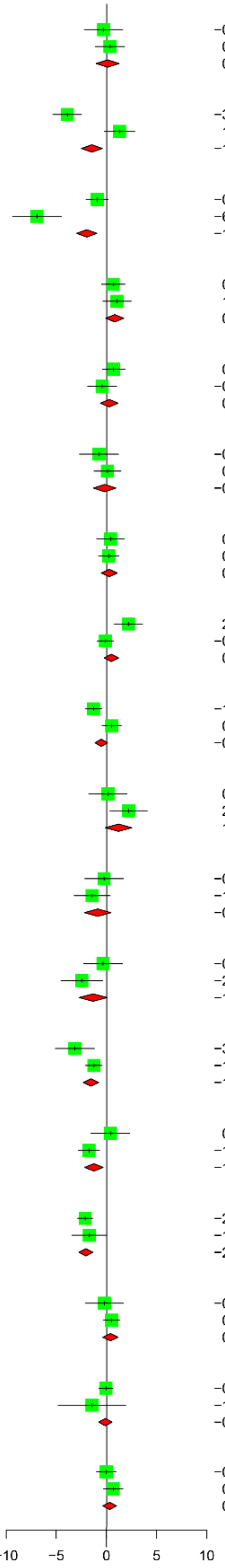

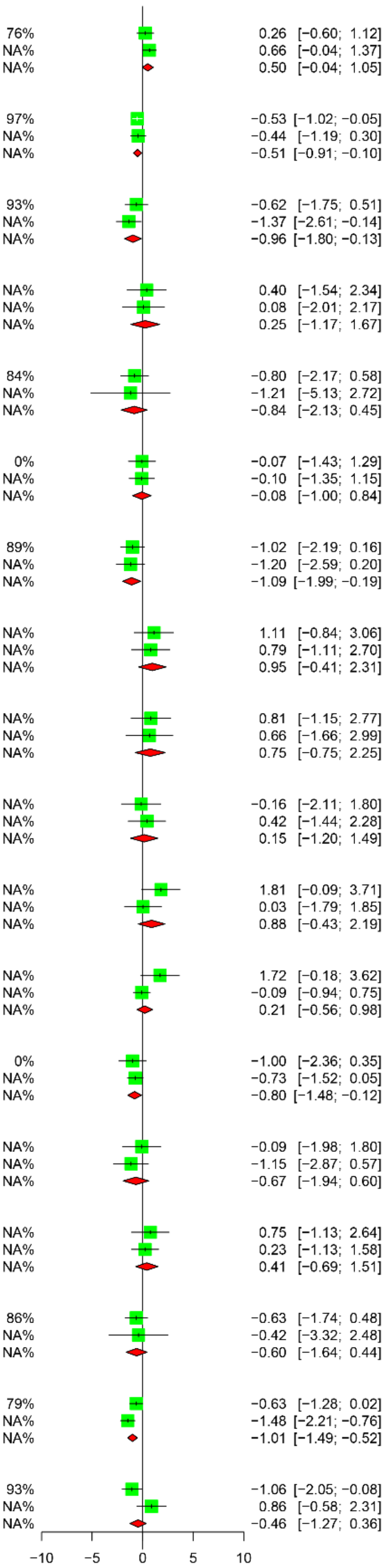

Comparison	Number of Studies	Direct Evidence	I2	SMD	95%−CI
**BoNTA+PT vs DxTP+PT**					
Direct estimate	1	0.37	NA%	−0.32	[−2.24; 1.60]
Indirect estimate			NA%	0.33	[−1.14; 1.79]
Network estimate			NA%	0.09	[−1.07; 1.25]
**BoNTA+PT vs HA+PT**					
Direct estimate	2	0.53	58%	−3.94	[−5.38; −2.50]
Indirect estimate			NA%	1.27	[−0.25; 2.80]
Network estimate			NA%	−1.48	[−2.53; −0.44]
**BoNTA+PT vs PT**					
Direct estimate	3	0.83	94%	−0.95	[−2.06; 0.17]
Indirect estimate			NA%	−6.96	[−9.39; −4.53]
Network estimate			NA%	−1.99	[−3.00; −0.98]
**CS vs CS+PT**					
Direct estimate	3	0.59	0%	0.64	[−0.54; 1.82]
Indirect estimate			NA%	1.03	[−0.39; 2.45]
Network estimate			NA%	0.80	[−0.11; 1.71]
**CS vs PT**					
Direct estimate	3	0.62	95%	0.69	[−0.43; 1.81]
Indirect estimate			NA%	−0.46	[−1.90; 0.99]
Network estimate			NA%	0.26	[−0.63; 1.14]
**CS vs UC**					
Direct estimate	1	0.33	NA%	−0.77	[−2.73; 1.18]
Indirect estimate			NA%	0.08	[−1.29; 1.44]
Network estimate			NA%	−0.20	[−1.32; 0.92]
**CS+PT vs OZ+PT**					
Direct estimate	2	0.35	0%	0.38	[−1.00; 1.76]
Indirect estimate			NA%	0.19	[−0.81; 1.19]
Network estimate			NA%	0.26	[−0.55; 1.07]
**CS+PT vs PRP+PT**					
Direct estimate	2	0.26	72%	2.16	[0.74; 3.58]
Indirect estimate			NA%	−0.13	[−0.96; 0.71]
Network estimate			NA%	0.47	[−0.26; 1.19]
**CS+PT vs PT**					
Direct estimate	6	0.57	96%	−1.31	[−2.14; −0.49]
Indirect estimate			NA%	0.50	[−0.46; 1.46]
Network estimate			NA%	−0.54	[−1.17; 0.08]
**DxTP vs DxTP+PT**					
Direct estimate	1	0.49	NA%	0.12	[−1.80; 2.03]
Indirect estimate			NA%	2.18	[0.32; 4.05]
Network estimate			NA%	1.18	[−0.16; 2.51]
**DxTP vs PT**					
Direct estimate	1	0.47	NA%	−0.27	[−2.20; 1.66]
Indirect estimate			NA%	−1.45	[−3.26; 0.35]
Network estimate			NA%	−0.90	[−2.22; 0.42]
**DxTP vs UC**					
Direct estimate	1	0.53	NA%	−0.37	[−2.30; 1.56]
Indirect estimate			NA%	−2.49	[−4.56; −0.42]
Network estimate			NA%	−1.36	[−2.77; 0.06]
**DxTP+PT vs HA+PT**					
Direct estimate	1	0.16	NA%	−3.18	[−5.14; −1.21]
Indirect estimate			NA%	−1.28	[−2.12; −0.43]
Network estimate			NA%	−1.57	[−2.35; −0.80]
**DxTP+PT vs OZ+PT**					
Direct estimate	1	0.23	NA%	0.37	[−1.56; 2.30]
Indirect estimate			NA%	−1.76	[−2.81; −0.72]
Network estimate			NA%	−1.28	[−2.20; −0.36]
**DxTP+PT vs PT**					
Direct estimate	7	0.84	97%	−2.14	[−2.90; −1.39]
Indirect estimate			NA%	−1.73	[−3.48; 0.02]
Network estimate			NA%	−2.08	[−2.77; −1.39]
**HA vs HA+PT**					
Direct estimate	1	0.16	NA%	−0.23	[−2.12; 1.66]
Indirect estimate			NA%	0.49	[−0.35; 1.33]
Network estimate			NA%	0.37	[−0.39; 1.14]
**HA vs PT**					
Direct estimate	8	0.96	83%	−0.08	[−0.76; 0.61]
Indirect estimate			NA%	−1.48	[−4.86; 1.90]
Network estimate			NA%	−0.13	[−0.80; 0.54]
**HA+PT vs OZ+PT**					
Direct estimate	4	0.51	93%	−0.04	[−1.00; 0.92]
Indirect estimate			NA%	0.64	[−0.33; 1.62]
Network estimate			NA%	0.29	[−0.39; 0.98]
**HA+PT vs PRP+PT**					
Direct estimate	5	0.40	76%	0.26	[−0.60; 1.12]
Indirect estimate			NA%	0.66	[−0.04; 1.37]
Network estimate			NA%	0.50	[−0.04; 1.05]
**HA+PT vs PT**					
Direct estimate	16	0.70	97%	−0.53	[−1.02; −0.05]
Indirect estimate			NA%	−0.44	[−1.19; 0.30]
Network estimate			NA%	−0.51	[−0.91; −0.10]
**HA+PT vs UC**					
Direct estimate	3	0.55	93%	−0.62	[−1.75; 0.51]
Indirect estimate			NA%	−1.37	[−2.61; −0.14]
Network estimate			NA%	−0.96	[−1.80; −0.13]
**MSC vs MSC+PT**					
Direct estimate	1	0.54	NA%	0.40	[−1.54; 2.34]
Indirect estimate			NA%	0.08	[−2.01; 2.17]
Network estimate			NA%	0.25	[−1.17; 1.67]
**MSC vs PT**					
Direct estimate	2	0.89	84%	−0.80	[−2.17; 0.58]
Indirect estimate			NA%	−1.21	[−5.13; 2.72]
Network estimate			NA%	−0.84	[−2.13; 0.45]
**MSC+PT vs PRP+PT**					
Direct estimate	2	0.46	0%	−0.07	[−1.43; 1.29]
Indirect estimate			NA%	−0.10	[−1.35; 1.15]
Network estimate			NA%	−0.08	[−1.00; 0.84]
**MSC+PT vs PT**					
Direct estimate	3	0.59	89%	−1.02	[−2.19; 0.16]
Indirect estimate			NA%	−1.20	[−2.59; 0.20]
Network estimate			NA%	−1.09	[−1.99; −0.19]
**OZ vs OZ+PT**					
Direct estimate	1	0.49	NA%	1.11	[−0.84; 3.06]
Indirect estimate			NA%	0.79	[−1.11; 2.70]
Network estimate			NA%	0.95	[−0.41; 2.31]
**OZ vs PRP**					
Direct estimate	1	0.59	NA%	0.81	[−1.15; 2.77]
Indirect estimate			NA%	0.66	[−1.66; 2.99]
Network estimate			NA%	0.75	[−0.75; 2.25]
**OZ vs PT**					
Direct estimate	1	0.48	NA%	−0.16	[−2.11; 1.80]
Indirect estimate			NA%	0.42	[−1.44; 2.28]
Network estimate			NA%	0.15	[−1.20; 1.49]
**OZ+PT vs PRGF+PT**					
Direct estimate	1	0.48	NA%	1.81	[−0.09; 3.71]
Indirect estimate			NA%	0.03	[−1.79; 1.85]
Network estimate			NA%	0.88	[−0.43; 2.19]
**OZ+PT vs PRP+PT**					
Direct estimate	1	0.17	NA%	1.72	[−0.18; 3.62]
Indirect estimate			NA%	−0.09	[−0.94; 0.75]
Network estimate			NA%	0.21	[−0.56; 0.98]
**OZ+PT vs PT**					
Direct estimate	2	0.25	0%	−1.00	[−2.36; 0.35]
Indirect estimate			NA%	−0.73	[−1.52; 0.05]
Network estimate			NA%	−0.80	[−1.48; −0.12]
**PRGF+PT vs PRP+PT**					
Direct estimate	1	0.45	NA%	−0.09	[−1.98; 1.80]
Indirect estimate			NA%	−1.15	[−2.87; 0.57]
Network estimate			NA%	−0.67	[−1.94; 0.60]
**PRP vs PRP+PT**					
Direct estimate	1	0.34	NA%	0.75	[−1.13; 2.64]
Indirect estimate			NA%	0.23	[−1.13; 1.58]
Network estimate			NA%	0.41	[−0.69; 1.51]
**PRP vs PT**					
Direct estimate	3	0.87	86%	−0.63	[−1.74; 0.48]
Indirect estimate			NA%	−0.42	[−3.32; 2.48]
Network estimate			NA%	−0.60	[−1.64; 0.44]
**PRP+PT vs PT**					
Direct estimate	9	0.56	79%	−0.63	[−1.28; 0.02]
Indirect estimate			NA%	−1.48	[−2.21; −0.76]
Network estimate			NA%	−1.01	[−1.49; −0.52]
**PT vs UC**					
Direct estimate	4	0.68	93%	−1.06	[−2.05; −0.08]
Indirect estimate			NA%	0.86	[−0.58; 2.31]
Network estimate			NA%	−0.46	[−1.27; 0.36]

Supplementary Figure S2. Forest plot summarizing the effects of treatment regimens on pain reduction for each follow-up timeframe

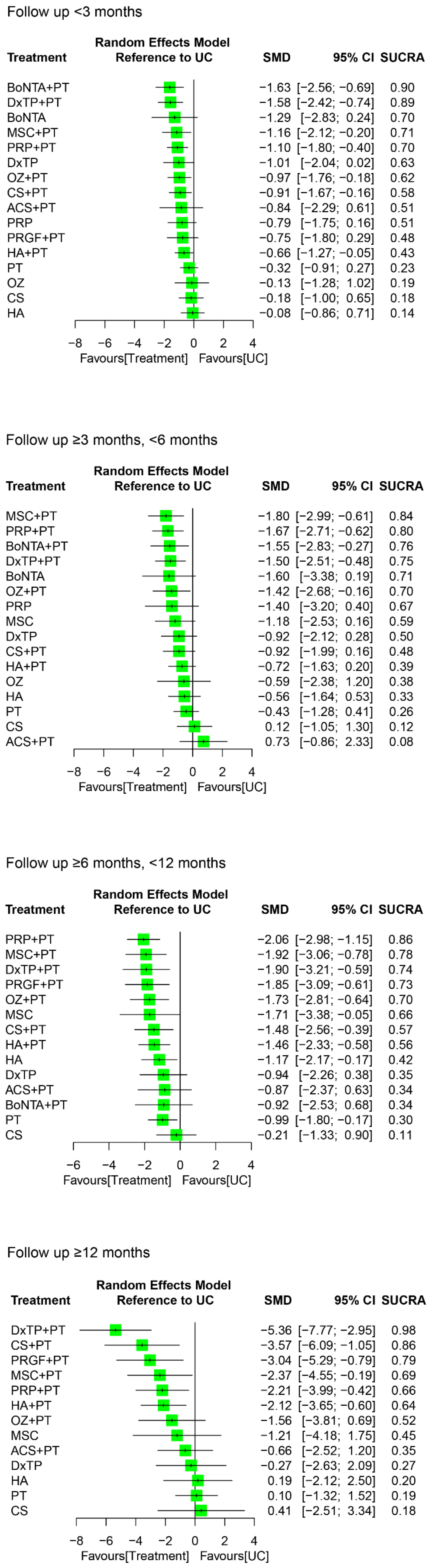

Supplementary Figure S3. Forest plot of node-splitting results for global function

Comparison	Number of Studies	Direct Evidence	I2	SMD	95%−CI
**BoNTA+PT vs DxTP+PT**					
Direct estimate	1	0.36	NA%	−0.10	[−1.53; 1.34]
Indirect estimate			NA%	−0.21	[−1.29; 0.88]
Network estimate			NA%	−0.17	[−1.03; 0.70]
**BoNTA+PT vs HA+PT**					
Direct estimate	2	0.56	0%	1.04	[0.00; 2.08]
Indirect estimate			NA%	0.40	[−0.76; 1.56]
Network estimate			NA%	0.76	[−0.02; 1.53]
**BoNTA+PT vs PT**					
Direct estimate	3	0.80	95%	1.18	[0.33; 2.02]
Indirect estimate			NA%	1.19	[−0.48; 2.86]
Network estimate			NA%	1.18	[0.43; 1.93]
**CS vs CS+PT**					
Direct estimate	3	0.60	66%	−0.47	[−1.39; 0.45]
Indirect estimate			NA%	−1.64	[−2.76; −0.53]
Network estimate			NA%	−0.94	[−1.65; −0.23]
**CS vs PT**					
Direct estimate	3	0.62	97%	−1.70	[−2.57; −0.82]
Indirect estimate			NA%	0.36	[−0.76; 1.47]
Network estimate			NA%	−0.91	[−1.60; −0.23]
**CS vs UC**					
Direct estimate	1	0.33	NA%	−0.16	[−1.64; 1.31]
Indirect estimate			NA%	0.12	[−0.92; 1.16]
Network estimate			NA%	0.02	[−0.83; 0.87]
**CS+PT vs OZ+PT**					
Direct estimate	2	0.36	0%	−0.08	[−1.12; 0.96]
Indirect estimate			NA%	−0.38	[−1.17; 0.40]
Network estimate			NA%	−0.27	[−0.90; 0.35]
**CS+PT vs PRP+PT**					
Direct estimate	2	0.27	97%	−2.00	[−3.10; −0.89]
Indirect estimate			NA%	−0.61	[−1.28; 0.06]
Network estimate			NA%	−0.98	[−1.55; −0.41]
**CS+PT vs PT**					
Direct estimate	5	0.52	89%	0.68	[−0.02; 1.39]
Indirect estimate			NA%	−0.69	[−1.43; 0.05]
Network estimate			NA%	0.03	[−0.48; 0.54]
**DxTP vs DxTP+PT**					
Direct estimate	1	0.49	NA%	0.58	[−0.86; 2.01]
Indirect estimate			NA%	−1.43	[−2.83; −0.03]
Network estimate			NA%	−0.45	[−1.45; 0.55]
**DxTP vs PT**					
Direct estimate	1	0.47	NA%	0.52	[−0.93; 1.97]
Indirect estimate			NA%	1.22	[−0.13; 2.57]
Network estimate			NA%	0.89	[−0.09; 1.88]
**DxTP vs UC**					
Direct estimate	1	0.54	NA%	0.62	[−0.83; 2.08]
Indirect estimate			NA%	3.22	[1.66; 4.78]
Network estimate			NA%	1.83	[0.77; 2.90]
**DxTP+PT vs HA+PT**					
Direct estimate	1	0.16	NA%	1.10	[−0.34; 2.55]
Indirect estimate			NA%	0.89	[0.26; 1.51]
Network estimate			NA%	0.92	[0.35; 1.50]
**DxTP+PT vs OZ+PT**					
Direct estimate	1	0.22	NA%	−0.01	[−1.46; 1.44]
Indirect estimate			NA%	1.35	[0.57; 2.13]
Network estimate			NA%	1.05	[0.36; 1.73]
**DxTP+PT vs PT**					
Direct estimate	7	0.85	94%	1.53	[0.97; 2.08]
Indirect estimate			NA%	0.33	[−0.97; 1.64]
Network estimate			NA%	1.34	[0.83; 1.86]
**HA vs HA+PT**					
Direct estimate	1	0.17	NA%	−0.02	[−1.41; 1.38]
Indirect estimate			NA%	−0.18	[−0.81; 0.45]
Network estimate			NA%	−0.15	[−0.73; 0.42]
**HA vs PT**					
Direct estimate	8	0.96	83%	0.26	[−0.26; 0.77]
Indirect estimate			NA%	0.58	[−1.91; 3.08]
Network estimate			NA%	0.27	[−0.23; 0.77]
**HA+PT vs OZ+PT**					
Direct estimate	4	0.51	83%	0.16	[−0.56; 0.88]
Indirect estimate			NA%	0.08	[−0.65; 0.81]
Network estimate			NA%	0.12	[−0.39; 0.64]
**HA+PT vs PRGF+PT**					
Direct estimate	2	0.82	0%	−0.99	[−1.99; −0.00]
Indirect estimate			NA%	−0.85	[−2.97; 1.27]
Network estimate			NA%	−0.97	[−1.87; −0.07]

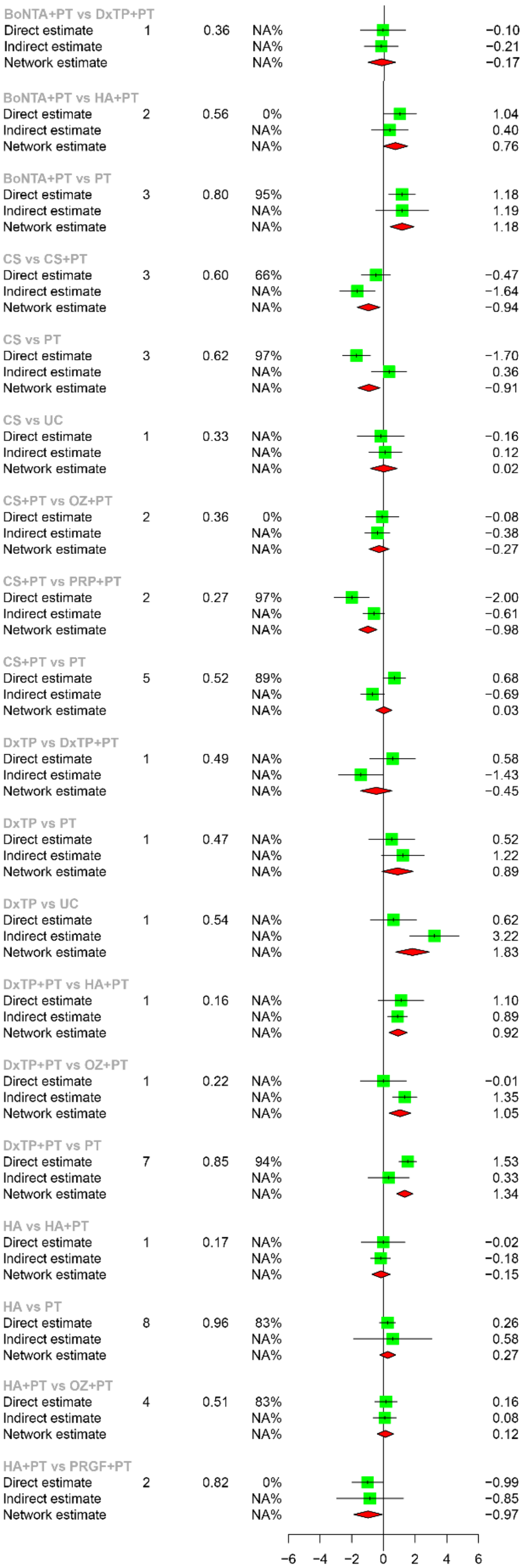

Comparison	Number of Studies	Direct Evidence	I2	SMD	95%−CI
**HA+PT vs PRP+PT**					
Direct estimate	5	0.41	90%	−0.53	[−1.17; 0.11]
Indirect estimate			NA%	−0.63	[−1.16; −0.09]
Network estimate			NA%	−0.59	[−1.00; −0.18]
**HA+PT vs PT**					
Direct estimate	16	0.71	84%	0.47	[0.11; 0.83]
Indirect estimate			NA%	0.31	[−0.25; 0.87]
Network estimate			NA%	0.42	[0.12; 0.72]
**HA+PT vs UC**					
Direct estimate	3	0.55	94%	1.16	[0.31; 2.02]
Indirect estimate			NA%	1.60	[0.66; 2.54]
Network estimate			NA%	1.36	[0.73; 1.99]
**MSC vs MSC+PT**					
Direct estimate	1	0.54	NA%	−0.12	[−1.58; 1.34]
Indirect estimate			NA%	−0.43	[−2.01; 1.16]
Network estimate			NA%	−0.26	[−1.34; 0.81]
**MSC vs PT**					
Direct estimate	2	0.89	50%	0.71	[−0.32; 1.74]
Indirect estimate			NA%	1.77	[−1.20; 4.74]
Network estimate			NA%	0.83	[−0.15; 1.80]
**MSC+PT vs PRP+PT**					
Direct estimate	2	0.48	22%	0.22	[−0.80; 1.24]
Indirect estimate			NA%	−0.04	[−1.02; 0.93]
Network estimate			NA%	0.08	[−0.62; 0.79]
**MSC+PT vs PT**					
Direct estimate	3	0.56	76%	0.94	[0.02; 1.86]
Indirect estimate			NA%	1.28	[0.23; 2.33]
Network estimate			NA%	1.09	[0.40; 1.78]
**OZ vs OZ+PT**					
Direct estimate	1	0.49	NA%	0.31	[−1.14; 1.77]
Indirect estimate			NA%	−0.25	[−1.69; 1.19]
Network estimate			NA%	0.03	[−0.99; 1.05]
**OZ vs PRP**					
Direct estimate	1	0.58	NA%	−0.68	[−2.16; 0.80]
Indirect estimate			NA%	−0.20	[−1.94; 1.54]
Network estimate			NA%	−0.48	[−1.61; 0.65]
**OZ vs PT**					
Direct estimate	1	0.47	NA%	0.09	[−1.39; 1.57]
Indirect estimate			NA%	0.54	[−0.85; 1.94]
Network estimate			NA%	0.33	[−0.69; 1.34]
**OZ+PT vs PRGF+PT**					
Direct estimate	1	0.48	NA%	−1.33	[−2.73; 0.08]
Indirect estimate			NA%	−0.88	[−2.22; 0.47]
Network estimate			NA%	−1.09	[−2.07; −0.12]
**OZ+PT vs PRP+PT**					
Direct estimate	1	0.17	NA%	−1.22	[−2.62; 0.19]
Indirect estimate			NA%	−0.60	[−1.24; 0.03]
Network estimate			NA%	−0.71	[−1.29; −0.13]
**OZ+PT vs PT**					
Direct estimate	2	0.25	36%	0.58	[−0.43; 1.60]
Indirect estimate			NA%	0.20	[−0.39; 0.79]
Network estimate			NA%	0.30	[−0.21; 0.81]
**PRGF+PT vs PRP+PT**					
Direct estimate	1	0.46	NA%	0.11	[−1.29; 1.51]
Indirect estimate			NA%	0.61	[−0.66; 1.89]
Network estimate			NA%	0.38	[−0.56; 1.33]
**PRP vs PRP+PT**					
Direct estimate	1	0.35	NA%	−0.21	[−1.60; 1.18]
Indirect estimate			NA%	−0.20	[−1.23; 0.83]
Network estimate			NA%	−0.20	[−1.03; 0.62]
**PRP vs PT**					
Direct estimate	3	0.87	90%	0.65	[−0.19; 1.48]
Indirect estimate			NA%	1.86	[−0.30; 4.02]
Network estimate			NA%	0.81	[0.03; 1.59]
**PRP+PT vs PT**					
Direct estimate	9	0.55	89%	0.80	[0.30; 1.30]
Indirect estimate			NA%	1.26	[0.71; 1.81]
Network estimate			NA%	1.01	[0.64; 1.38]
**PT vs UC**					
Direct estimate	4	0.66	95%	1.70	[0.94; 2.46]
Indirect estimate			NA%	−0.55	[−1.61; 0.52]
Network estimate			NA%	0.94	[0.32; 1.56]

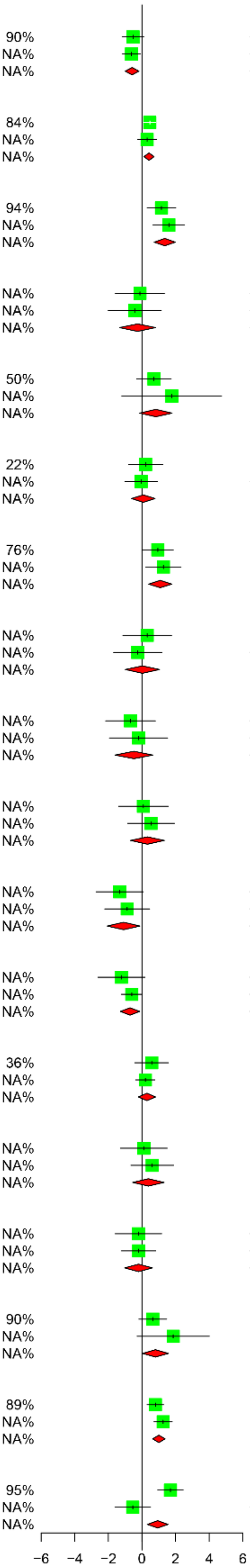

Supplementary Figure S4. Forest plot summarizing the effects of treatment regimens on global function recovery for each follow-up time frame.

Follow up <3 months

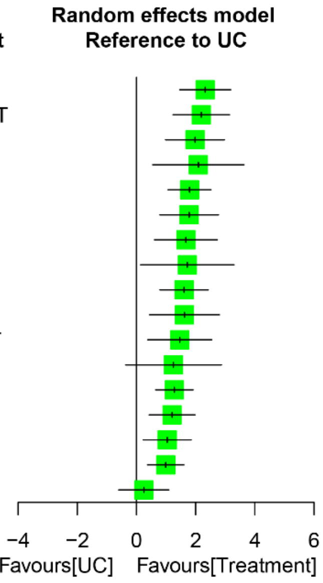

Treatment	SMD	95% CI	SUCRA
DxTP+PT	2.32	[1.46; 3.19]	0.89
BoNTA+PT	2.19	[1.23; 3.15]	0.83
MSC+PT	1.98	[0.98; 2.97]	0.74
ACS+PT	2.09	[0.54; 3.64]	0.73
PRP+PT	1.79	[1.06; 2.52]	0.66
PRP	1.78	[0.79; 2.77]	0.65
DxTP	1.67	[0.60; 2.74]	0.59
BoNTA	1.72	[0.13; 3.30]	0.58
OZ+PT	1.61	[0.79; 2.43]	0.57
OZ	1.62	[0.44; 2.81]	0.56
PRGF+PT	1.46	[0.38; 2.55]	0.49
SVF+PT	1.25	[−0.37; 2.87]	0.40
HA+PT	1.28	[0.64; 1.92]	0.38
CS+PT	1.20	[0.42; 1.98]	0.34
HA	1.04	[0.22; 1.85]	0.26
PT	0.99	[0.37; 1.61]	0.21
CS	0.25	[−0.60; 1.10]	0.06

Follow up ≥3 months, <6 months

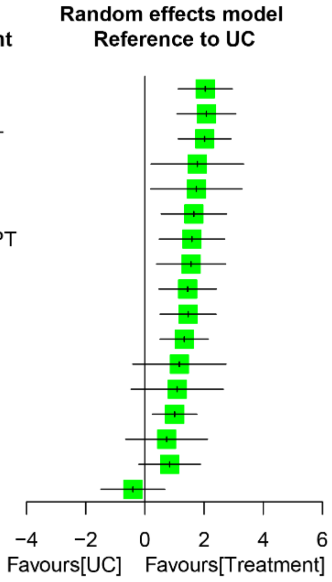

Treatment	SMD	95% CI	SUCRA
PRP+PT	2.04	[1.12; 2.96]	0.84
MSC+PT	2.08	[1.08; 3.07]	0.83
DxTP+PT	2.02	[1.12; 2.91]	0.82
PRP	1.77	[0.20; 3.34]	0.67
BoNTA	1.74	[0.20; 3.28]	0.65
OZ+PT	1.65	[0.54; 2.76]	0.65
BoNTA+PT	1.59	[0.48; 2.70]	0.62
MSC	1.56	[0.39; 2.73]	0.59
CS+PT	1.44	[0.47; 2.41]	0.54
HA	1.46	[0.51; 2.41]	0.54
HA+PT	1.33	[0.51; 2.14]	0.47
SVF+PT	1.16	[−0.42; 2.74]	0.44
OZ	1.09	[−0.47; 2.65]	0.39
PT	1.00	[0.25; 1.76]	0.29
ACS+PT	0.73	[−0.65; 2.12]	0.28
DxTP	0.84	[−0.21; 1.88]	0.27
CS	−0.41	[−1.49; 0.67]	0.03

Follow up ≥6 months, <12 months

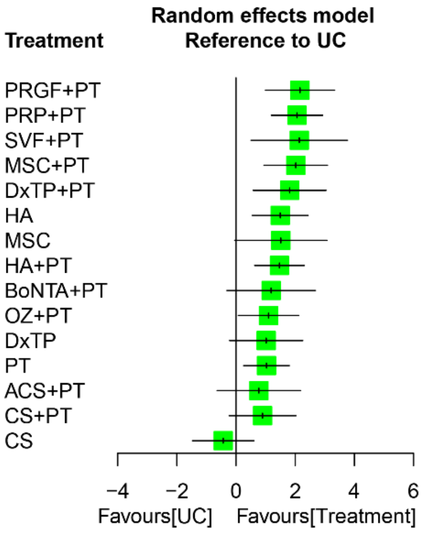

Treatment	SMD	95% CI	SUCRA
PRGF+PT	2.16	[0.99; 3.33]	0.84
PRP+PT	2.06	[1.19; 2.93]	0.83
SVF+PT	2.14	[0.51; 3.77]	0.80
MSC+PT	2.02	[0.94; 3.09]	0.79
DxTP+PT	1.81	[0.58; 3.05]	0.72
HA	1.49	[0.55; 2.44]	0.58
MSC	1.51	[−0.05; 3.08]	0.57
HA+PT	1.47	[0.63; 2.31]	0.56
BoNTA+PT	1.19	[−0.31; 2.69]	0.45
OZ+PT	1.10	[0.07; 2.12]	0.38
DxTP	1.02	[−0.22; 2.26]	0.37
PT	1.03	[0.25; 1.80]	0.33
ACS+PT	0.77	[−0.64; 2.18]	0.32
CS+PT	0.90	[−0.24; 2.03]	0.31
CS	−0.43	[−1.48; 0.61]	0.03

Follow up ≥12 months

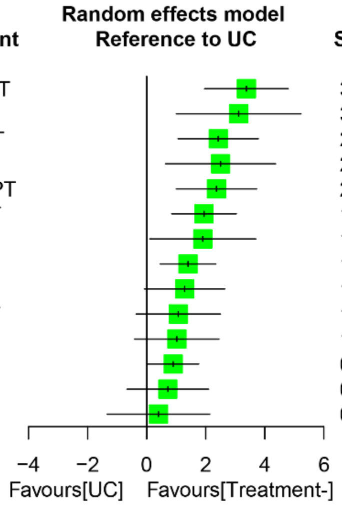

Treatment	SMD	95% CI	SUCRA
DxTP+PT	3.38	[1.96; 4.80]	0.93
CS+PT	3.11	[1.00; 5.23]	0.86
MSC+PT	2.42	[1.06; 3.78]	0.76
SVF+PT	2.50	[0.63; 4.37]	0.75
PRGF+PT	2.36	[1.00; 3.73]	0.75
PRP+PT	1.94	[0.84; 3.04]	0.63
MSC	1.90	[0.10; 3.70]	0.60
HA+PT	1.40	[0.45; 2.35]	0.45
OZ+PT	1.29	[−0.08; 2.65]	0.40
ACS+PT	1.07	[−0.36; 2.50]	0.34
DxTP	1.02	[−0.41; 2.45]	0.32
PT	0.90	[0.03; 1.77]	0.26
HA	0.72	[−0.67; 2.10]	0.23
CS	0.40	[−1.34; 2.14]	0.18

Supplementary Figure S5. Forest plot of node-splitting results for walking capability

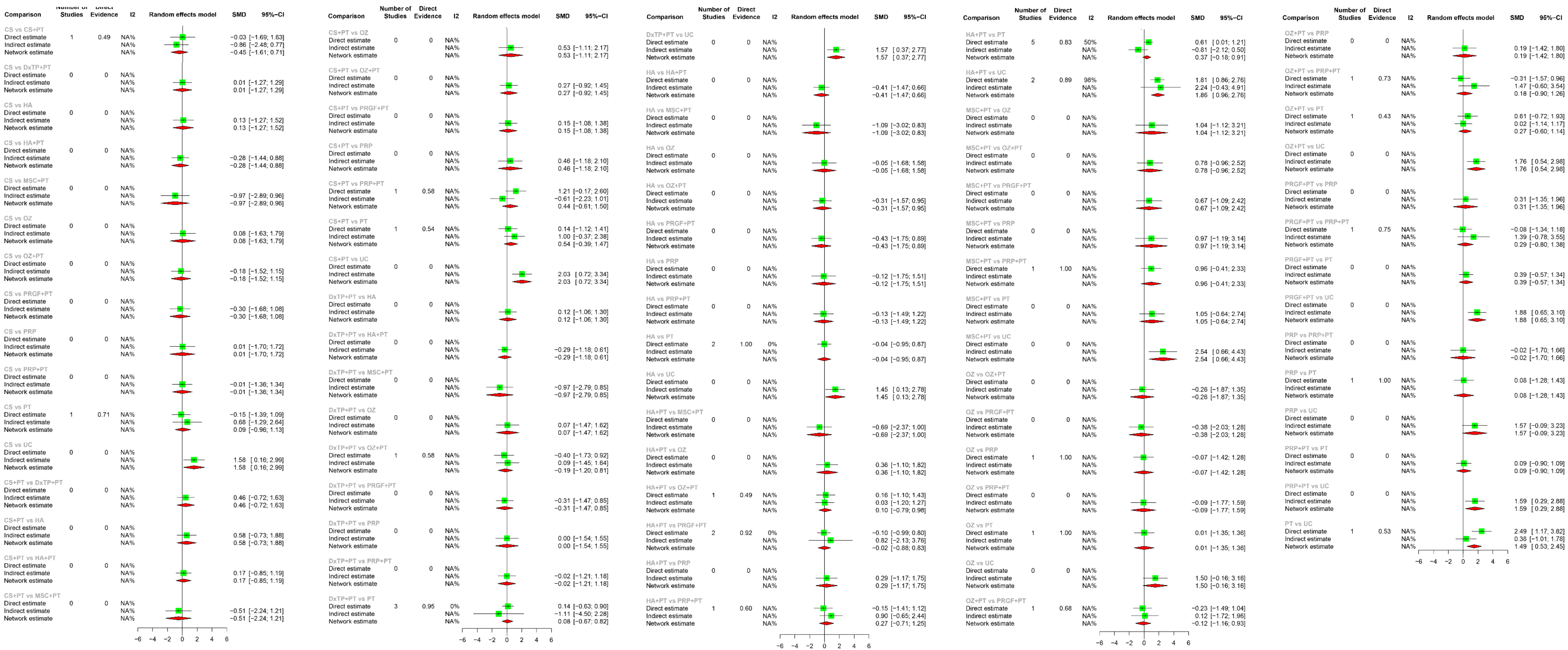

Supplementary Figure S6. Forest plot summarizing the effects of treatment regimens on walking capability restoration for each follow-up time frame

Follow up <3 months

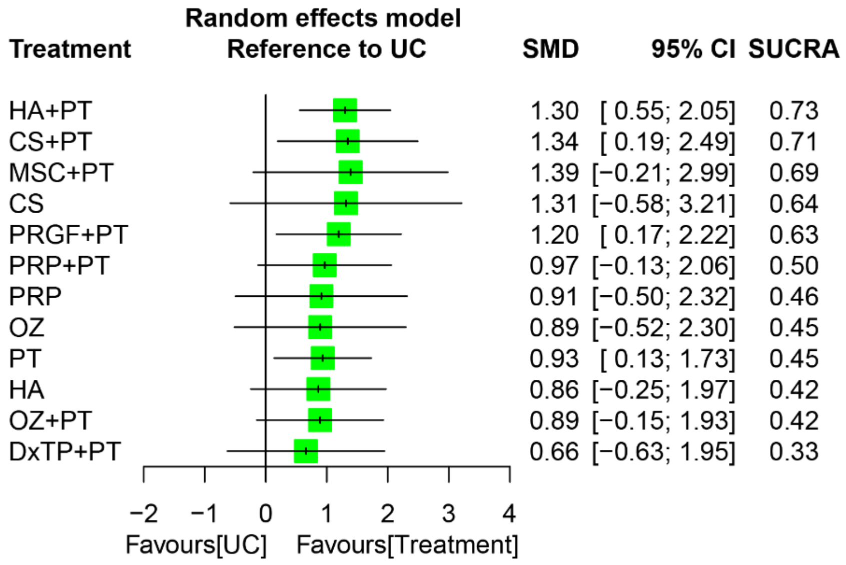

Follow up ≥3 months, <6 months

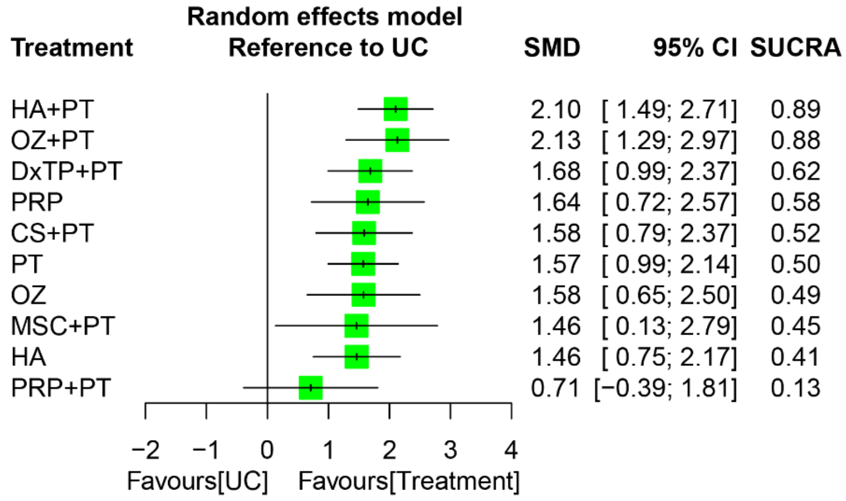

Follow up ≥6 months, <12 months

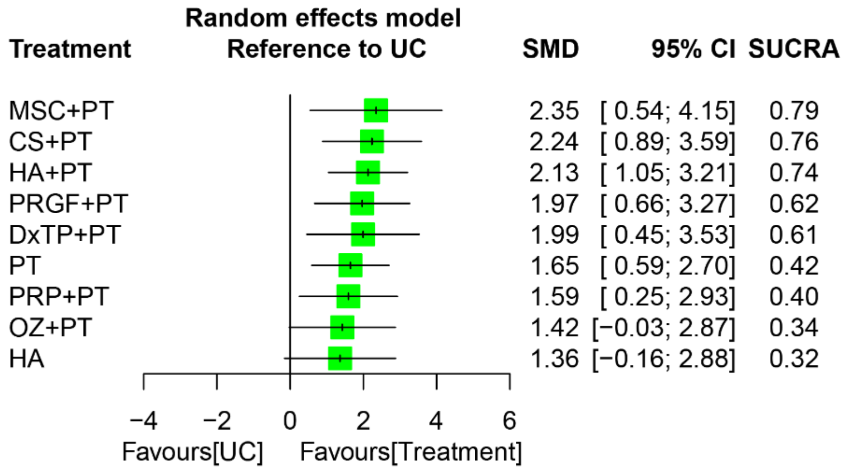

Follow up ≥12 months

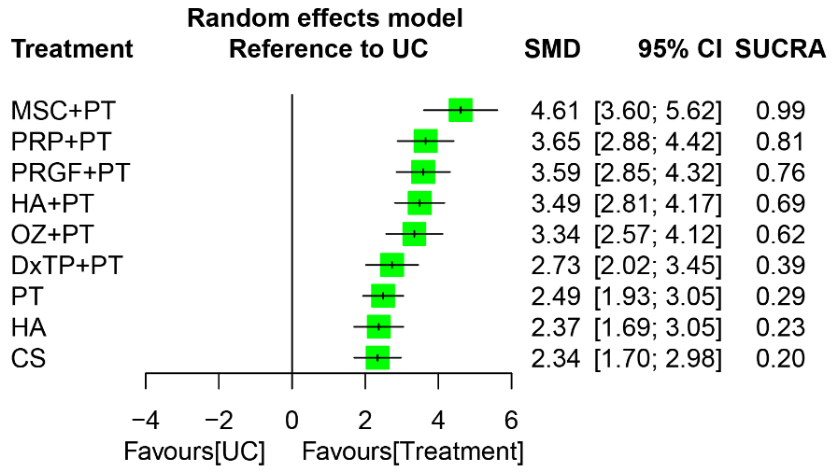

Supplementary Figure S7. Funnel plot representing publication bias for pain (A), global function (B), and walking capability (C)

(A)

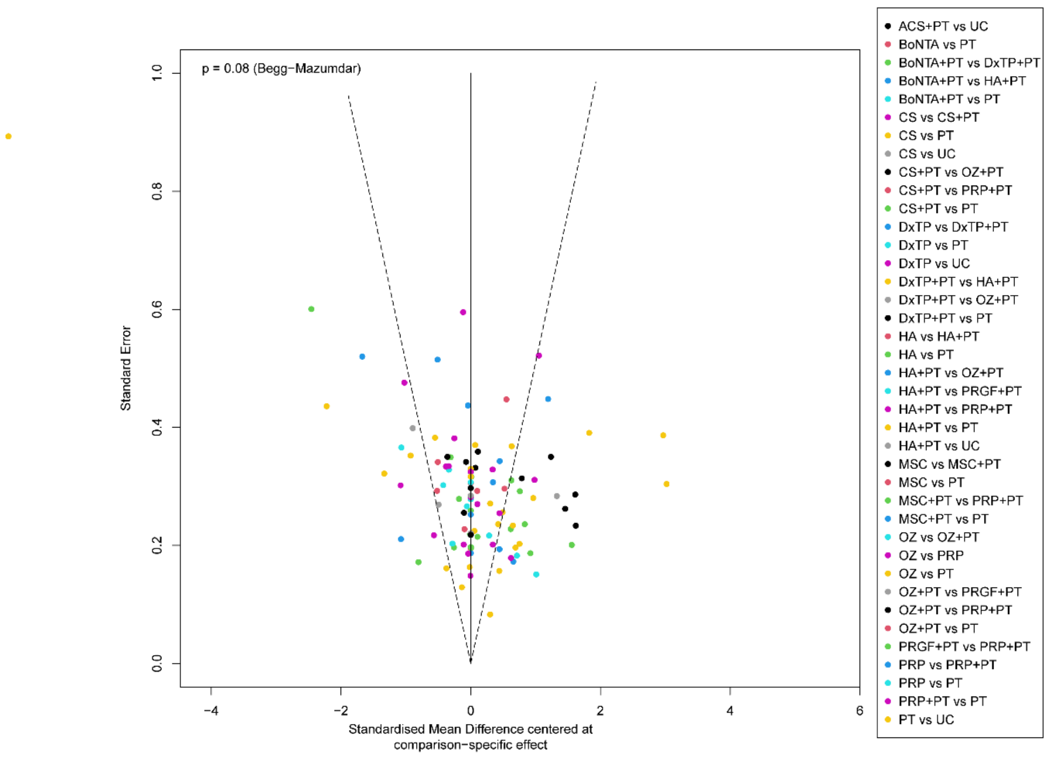

(B)

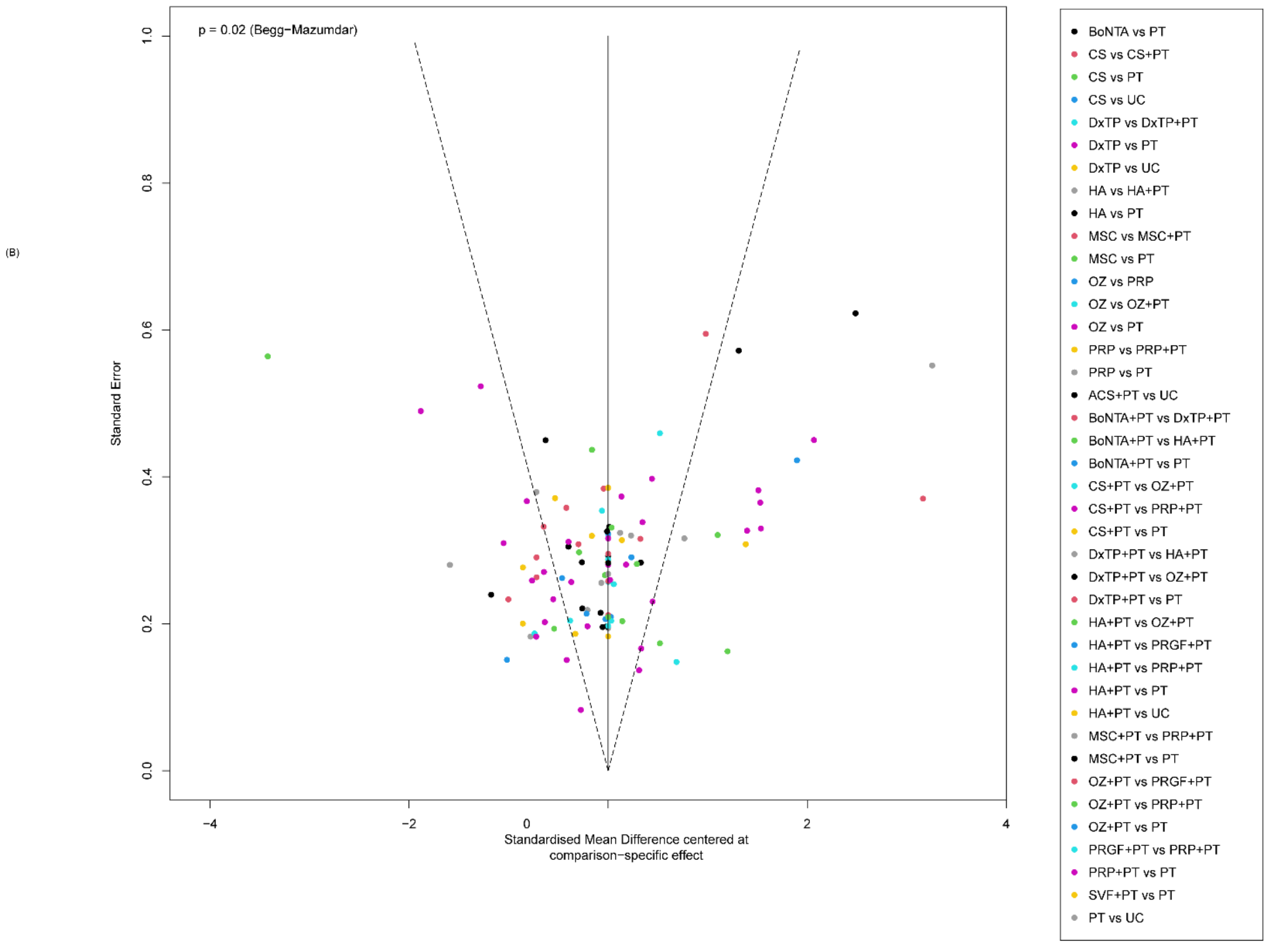

(C)

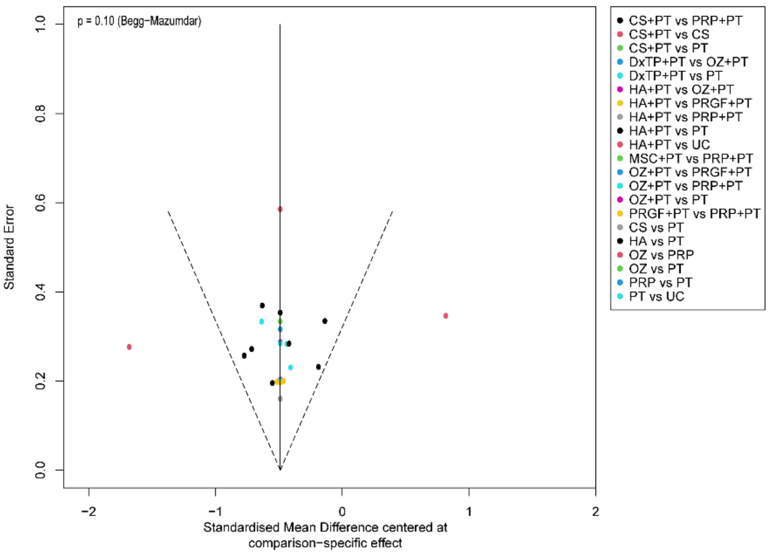

Supplement: Supplementary file 1 [file ijms-24-06078-s001.zip › ijms-2274985-supplementary.pdf]
